# The *Toxoplasma gondii* Cyst Wall Protein CST1 Is Critical for Cyst Wall Integrity and Promotes Bradyzoite Persistence

**DOI:** 10.1371/journal.ppat.1003823

**Published:** 2013-12-26

**Authors:** Tadakimi Tomita, David J. Bzik, Yan Fen Ma, Barbara A. Fox, Lye Meng Markillie, Ronald C. Taylor, Kami Kim, Louis M. Weiss

**Affiliations:** 1 Department of Pathology, Albert Einstein College of Medicine, Bronx, New York, New York, United States of America; 2 Department of Microbiology and Immunology, Geisel School of Medicine at Dartmouth, Lebanon, New Hampshire, United States of America; 3 Fundamental and Computational Sciences, Pacific Northwest National Laboratory, Richland, Washington, United States of America; 4 Department of Medicine, Albert Einstein College of Medicine, Bronx, New York, New York, United States of America; 5 Department of Microbiology and Immunology, Albert Einstein College of Medicine, Bronx, New York, New York, United States of America; University of Geneva, Switzerland

## Abstract

*Toxoplasma gondii* infects up to one third of the world's population. A key to the success of *T. gondii* as a parasite is its ability to persist for the life of its host as bradyzoites within tissue cysts. The glycosylated cyst wall is the key structural feature that facilitates persistence and oral transmission of this parasite. Because most of the antibodies and reagents that recognize the cyst wall recognize carbohydrates, identification of the components of the cyst wall has been technically challenging. We have identified CST1 (TGME49_064660) as a 250 kDa SRS (SAG1 related sequence) domain protein with a large mucin-like domain. CST1 is responsible for the *Dolichos biflorus* Agglutinin (DBA) lectin binding characteristic of *T. gondii* cysts. Deletion of *CST1* results in reduced cyst number and a fragile brain cyst phenotype characterized by a thinning and disruption of the underlying region of the cyst wall. These defects are reversed by complementation of *CST1*. Additional complementation experiments demonstrate that the CST1-mucin domain is necessary for the formation of a normal cyst wall structure, the ability of the cyst to resist mechanical stress, and binding of DBA to the cyst wall. RNA-seq transcriptome analysis demonstrated dysregulation of bradyzoite genes within the various *cst1* mutants. These results indicate that CST1 functions as a key structural component that confers essential sturdiness to the *T. gondii* tissue cyst critical for persistence of bradyzoite forms.

## Author Summary

*Toxoplasma gondii* causes severe encephalitis in immune compromised hosts after reactivation of brain cysts that persist for the life span of the host. The biological mechanisms of bradyzoite persistence within cysts are not fully understood. The glycosylated cyst wall is thought to play a crucial role in survival of bradyzoites during chronic infection as well as successful oral transmission of infection. Here we have identified the gene encoding cyst wall glycoprotein CST1. When we delete the CST1 gene, parasites form dramatically fragile brain cysts. Parasites lacking CST1 develop fewer brain cysts, show dysregulation of bradyzoite-specific gene expression and are less able to grow under stressed conditions. The rescue of these phenotypes requires the heavily glycosylated mucin domain of CST1. These studies demonstrate that the glycosylation of CST1 plays a significant role in the structural integrity and persistence of brain cysts. Agents that perturb CST1 glycosylation have the potential to disrupt formation of latent brain cysts, preventing chronic *Toxoplasma* infection.

## Introduction

*Toxoplasma gondii*, an Apicomplexan, is an obligate intracellular protozoan parasite that can cause severe human disease. It is estimated that a third of the human population is chronically infected with *T. gondii* [1], with prevalence rates ranging from a few percent to nearly 80% depending on the population [2]. This parasite can cause lethal encephalitis in immune compromised individuals such as those with AIDS or organ transplant recipients on immune suppressive medications. It is also the cause of a devastating congenital disease, which may result in blindness and mental retardation if infection occurs in a *T. gondii* seronegative pregnant woman. During acute infection, the parasites proliferate as the fast-growing tachyzoite life cycle form, which causes a disseminated systemic infection. This disseminated acute infection is controlled by interferon-γ and T cell responses. In response to stress signals during acute infection, such as the immune response or programmed spontaneous differentiation responses, tachyzoites differentiate into the slow-growing bradyzoite life cycle stage that remains latent in the host. Bradyzoites can form tissue cysts in brain, muscles and visceral organs and when tissue cysts are orally ingested the released bradyzoites differentiate into tachyzoites, causing an acute infection in a new host. Bradyzoite differentiation processes and the development and maintenance of tissue cysts are critical for transmission of *T. gondii* infection. Evidence suggests that the latent tissue cysts evade the immune response [3] and can persist for the host life span [4]. It is likely tissue cysts occasionally rupture and any released parasites [5] are cleared by immune system. In the absence of an effective immune response these released organisms can differentiate into tachyzoites causing an acute infection. Thus, tissue cysts serve as reservoir for the reactivation of the toxoplasmosis when the host becomes immune compromised with conditions such as AIDS or organ transplantation.

Tissue cysts can range from 5 to 100 µm in size containing just a few to thousands of encysted bradyzoites. Tissue cysts can be found in any organ, but are especially prevalent in the central nervous system. The bradyzoites within the tissue cyst are covered by a prominent translucent 0.25 to 0.75 µm thick cyst wall structure (cyst wall) which can be visualized using electron microscopy [6]. The cyst wall forms beneath a modified parasitophorous vacuole membrane containing bradyzoites. The cyst wall is highly glycosylated and stains easily with periodic acid-Schiff, *Dolichos biflorus* lectin (DBA), and succinylated wheat germ agglutinin [6]–[8]. These carbohydrate modifications of the cyst wall are hypothesized to mask cyst wall proteins from host immune responses and to provide structural and chemical resistance against environmental stress, facilitating transmission of this pathogen [9].

The biogenesis, composition, and functions of the cyst wall are not yet well defined. A cyst wall glycoprotein CST1 was discovered more than a decade ago [10]. This protein, CST1, binds to DBA lectin, suggesting that it is a glycoprotein that contains N-acetyl-galactosamine. CST1 localized to the *in vivo* and *in vitro* cyst wall, but was not found associated with the tachyzoite parasitophorous vacuole. The corresponding gene, *CST1*, has not previously been identified, as the available monoclonal antibody 73.18 [11] to CST1 recognizes a glycoepitope and attempts to identify the glycoprotein recognized by this monoclonal antibody were unsuccessful (Weiss LM, unpublished). While some progress has occurred, cyst wall biology is still poorly understood despite the clinical and biological importance of this structure for transmission and latency in this important protozoan infection [12]. We produced a new monoclonal antibody library to *T. gondii* tissue cysts and used a combination of microscopic, genetic and proteomic approaches to identify cyst wall components. Using this approach we identified *CST1*, the gene corresponding to CST1, and characterized the effect of a knockout of this gene on *T. gondii*.

## Results

### Monoclonal antibody SalmonE binds to the cyst wall

**Figure 1:**
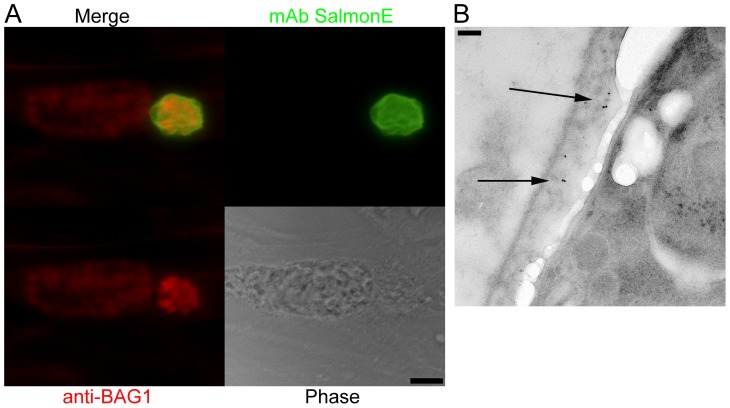
Monoclonal antibody SalmonE binds to the cyst wall of cysts isolated *in vitro* and *in vivo*. (**A**) Human foreskin fibroblasts (HFF) were infected with ME49 strain of *T. gondii* under alkaline conditions (pH 8.1) for 3 days. Anti-BAG1 antibody (red) staining demonstrates that the smaller parasitophorous vacuole on the right is an *in vitro* cyst; mAb SalmonE (green) binds to the parasitophorous vacuole of *in vitro* cyst, but not to the larger parasitophorous vacuole containing tachyzoites on the left. Bar, 10 µm. (**B**) Immuno-electron microscopic labeling of the cyst wall with mAb SalmonE. A ME49 brain tissue cyst isolated from an infected mouse was processed for immuno-electron microscopy and probed with mAb SalmonE. The gold particles demonstrate that mAb SalmonE localizes to the granular layer (arrow) under the limiting membrane of cyst wall. Bar, 200 nm.

To identify cyst wall proteins, a hybridoma library was created from mice immunized with a lysate of *T. gondii* ME49 cysts purified from the brains of mice with chronic *T. gondii* infection. From this library, we screened monoclonal antibodies by immunofluorescence against ME49 *T. gondii in vitro* cysts (bradyzoite-) and tachyzoite-containing vacuoles. Among the 189 cyst-wall positive hybridomas, we identified an mAb clone SalmonE that reacted with bradyzoite-containing parasitophorous vacuoles and uniformly stained the limiting parasitophorous vacuole membrane of BAG1-positive parasites (bradyzoites) but did not stain vacuoles containing BAG1 negative parasites (Figure 1A). BAG1 negative vacuoles were positive for SAG1, a tachyzoite specific marker (data not shown). This candidate cyst wall reactive monoclonal antibody was used to further characterize the cyst wall.

Since *in vitro* cysts do not completely differentiate, we examined the localization of SalmonE in cysts isolated from mice with chronic *T. gondii* infection. Within these more mature cysts there is a more organized cyst wall structure, and the bradyzoites within these cysts enter G_o_ and arrest in the cell cycle [12], [13]. *T. gondii* (ME49 strain) *in vivo* brain cysts harvested from infected mice were labeled with SalmonE and analyzed using immuno-electron microscopy (Figure 1B). SalmonE recognizes the diffuse thick layer of the cyst wall beneath the limiting membrane of the cyst wall in a distribution similar to the reactivity of the mAb specific for CST1 as well as the DBA lectin [10].

### CST1, the mAb SalmonE reactive molecule, is a SRS glycoprotein with an extended mucin domain

**Figure 2:**
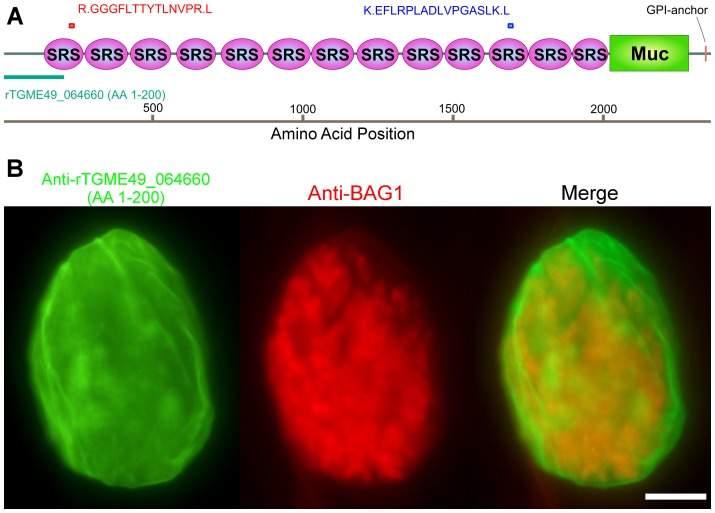
Monoclonal antibody SalmonE recognizes cyst wall protein CST1 (TGME49_064660). (**A**) The two peptides (red and blue bars) identified by mass spectrometry were mapped to the TGME49_064660 (CST1) protein. The recombinant protein rTGME49_064660 is the first 200 amino acids of the predicted gene (green bar). (**B**) CST1 (TGME49_064660) antiserum stains in vitro cyst walls. HFF cells were infected with ME49 strain of *T. gondii* under alkaline conditions for 3 days. Anti-rTGME49_064660 antiserum (mice immunized with TGME49_064660 recombinant protein green) stains the parasitophorous vacuole containing BAG1-positive parasites (red). Bar, 10 µm.

To determine the target of monoclonal antibody SalmonE, antigens from ME49 *in vitro* derived bradyzoite lysates were immunoprecipitated with mAb SalmonE, separated with SDS-PAGE and the two major candidate protein bands (a low signal intensity 150 kDa band and a high signal intensity high molecular weight band in the stacking gel) were excised and analyzed by MALDI-TOF mass spectrometry. The high molecular band had two peptides that matched the predicted gene product of TGME49_064660 (peptides: RGGGFLTTYTLNVPRL and KEFLRPLADLVPGASLKL, MASCOT, *p*<10^−7^, Figure 2A), which had been annotated as SRS44 in a published analysis of SRS domain-containing proteins [14]. The low molecular weight band had two peptides that matched SRS13 (peptides: KLPEKPAAAVAR and LTLDAGPPQATTLCYK), a glycoprotein we also subsequently characterized (Tomita and Weiss, in preparation). Polyclonal murine antiserum raised to the first 200 amino acids of SRS13 did not react with the cyst wall (data not shown). There was no other protein identified from SalmonE immunoprecipitated bands. To verify that the TGME49_064660 gene product is responsible for the cyst wall staining of monoclonal antibody SalmonE, mouse antiserum was raised against recombinant TGME49_064660 protein consisting of the first 200 amino acid of the predicted gene (Figure 2A, rTGME49_064660). Probing *in vitro* cysts with the anti-rTGME49_064660 serum revealed a similar pattern of staining as seen with the monoclonal antibody SalmonE (Figure 1A and 2B). This verified that TGME49_064660 is indeed a cyst wall gene. After completion of molecular verification (see below for details) TGME49_064660 was identified as *CST1*, the gene corresponding to the previously identified protein CST1 [10].

The cyst wall localization as well as its identification as an SRS protein suggested that CST1 should be a secreted protein; however, the current annotated TGME49_064660 gene product in ToxoDB.org does not contain a potential signal peptide sequence (SignalP 4.0 prediction [15]). Examination of the upstream sequences (Figure S1A), suggested that the gene model was incorrect. Use of 5′ rapid amplification of cDNA ends (RACE) demonstrated additional coding sequence, a revised 5′ UTR (Figure S1A) and also confirmed that *CST1* (TGME49_064660) does not extend into the predicted upstream gene TGME49_064670. Sequence analysis of the 5′ RACE product revealed an in-frame methionine codon located 43 residues upstream of the annotated predicted start site (Figure S1A). The protein predicted using this upstream methionine codon encodes a high probability signal peptide with a cleavage site; therefore, it is likely that CST1 translation begins at this methionine, 43 residues upstream of the annotated initiator methionine codon.

**Figure 3:**
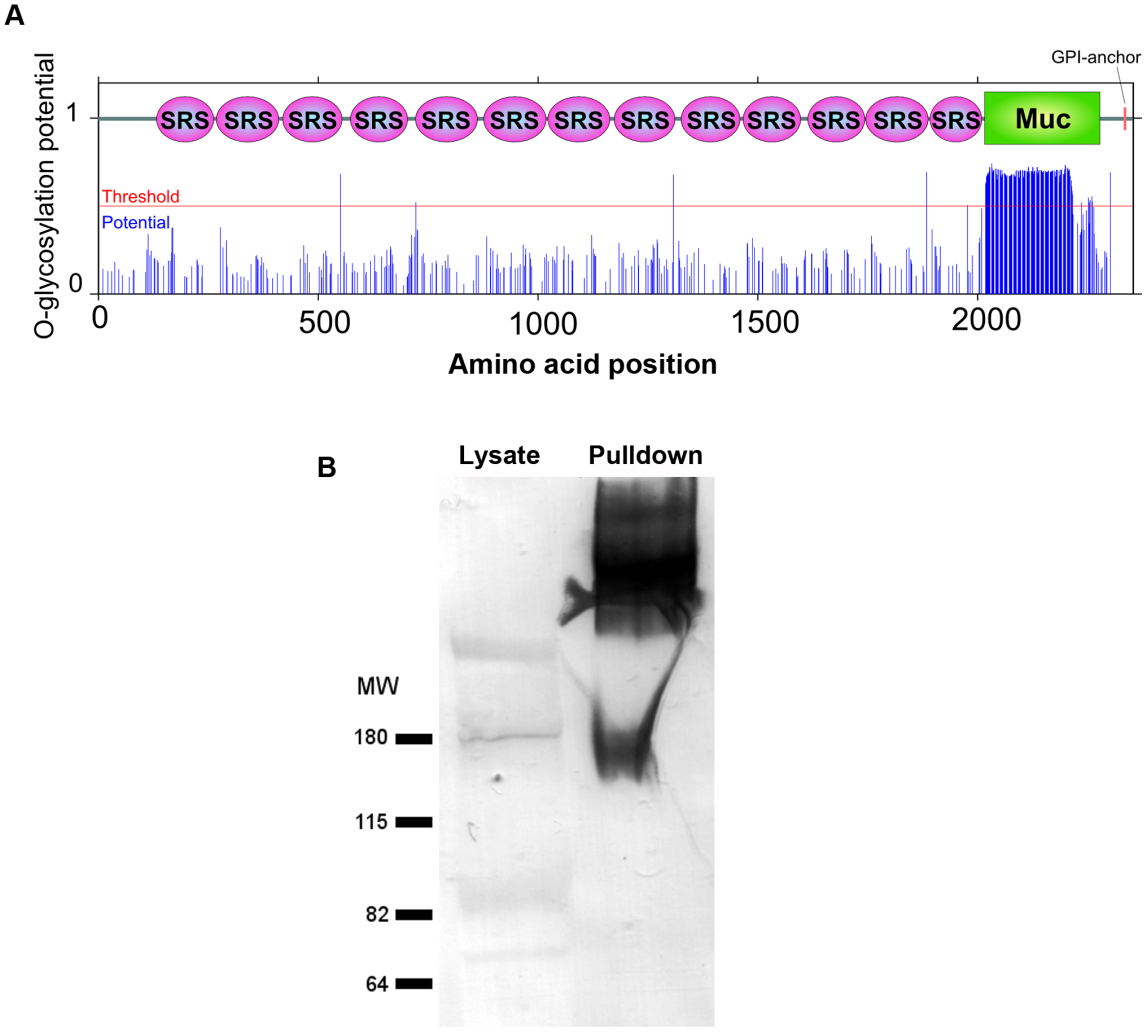
The mucin domain of CST1 is highly O-glycosylated. (**A**) The glycosylation potential of CST1 was predicted by the NetOGlyc 3.1. Red line indicates the threshold and blue line indicates the potential at each S/T amino acid position with domain-scheme overlay. This method predicts that the mucin domain is likely to be highly O-glycosylated. (**B**) mAb SalmonE immunoprecipitates are bound by DBA lectin. The left lane is 10 µl of whole parasite lysate (ME49 at pH 8.1) and the right lane is the sample (equivalent to 200 µl lysate) that was immunoprecipitated with mAb SalmonE, separated by SDS-PAGE and transferred to nitrocellulose membrane. The membrane was probed with AP-conjugated DBA lectin.

CST1 contains thirteen SRS domains (Figure 2A) and is unique among the SRS family proteins in having such a large number of SRS domains. Another striking feature of the predicted protein is the presence of a 263 amino acid stretch with multiple threonine-rich tandem repeats of T_5–11_[R/I]K_2_P; this region has homology to the mucin-like domains in a major glycoprotein of *Cryptosporidium parvum* (GP900, CMU_014140) (Figure 2A). Since mucin domains are typically extensively O-glycosylated on Ser or Thr residues, the probability of O-glycosylation at this mucin domain was assessed using neural network model NetOGlyc 3.1 [16]. Of the 157 threonines in the mucin domain, 95% were predicted to be O-glycosylated using NetOGlyc 3.1(Figure 3A).

To investigate whether the CST1 is O-glycosylated, SalmonE-immunoprecipitates were probed with *Dolichos biflorus* lectin (DBA), a marker for the cyst wall that recognizes GalNAc [17] (Figure 3B). DBA lectin overlays verified that CST1, the TGME49_064660 gene product, is a glycoprotein. *CST1* mRNA is expressed in type I (RH), type II (P/ME49) and type III (CTG) strains as evidenced by expression data from www.ToxoDB.org and our RNA-seq data (Figure S1).

### CST1 is not required for cyst formation

**Figure 4:**
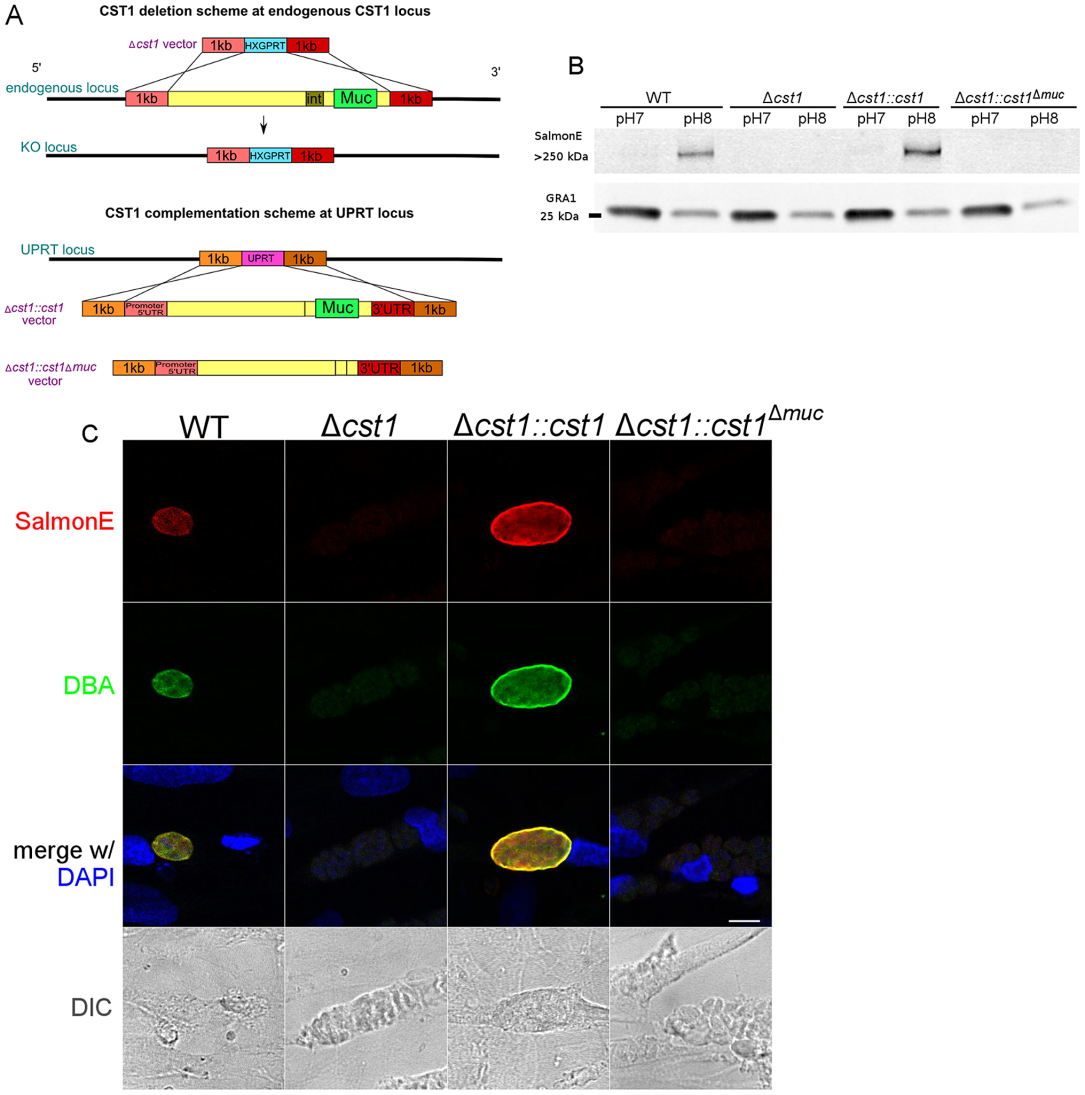
Characterization of CST1 knock out (Δ*cst1*) and CST1 complemented *T. gondii* strains. (**A**) Schematic representation of disruption of *CST1* and complementation of Δ*cst1* mutant. Upper half of the diagram represents the deletion of whole *CST1* gene with Δ*cst1* vector. Lower half represents the complementation of *CST1* genes at UPRT locus. (**B**) Immunoblot of *T. gondii* parasite cultures grown in normal and alkaline conditions probed with mAb SalmonE. SalmonE reactive antigen (CST1) is induced in alkaline conditions. Δ*cst1* knockout parasites are not recognized by mAb Salmon E, but reactivity is restored in full-length complement (Δ*cst1::cst1*) but CST1^Δmuc^ protein lacks the reactivity due to the loss of mucin-like domain. The parasite specific dense granule protein GRA1 is used as a loading control. (**C**) CST1 and DBA co-localize on *in vitro* cyst wall. HFF cells infected with WT, Δ*cst1*, Δ*cst1::cst1*, or Δ*cst1::cst1* ^Δ*muc*^ *T. gondii* strains under alkaline conditions were probed with SalmonE (red) or DBA lectin (green). Monoclonal antibody SalmonE and DBA staining co-localized. The presence of the full-length CST1 gene is necessary for mAb SalmonE and DBA staining on cyst wall. Bar, 10 µm.

To understand the function of CST1 we deleted the entire CST1 gene (Figure 4A) in the PruΔ*ku80* background [18]. This strain has a high frequency of homologous recombination that facilitates the development of knock-outs and also contains GFP under the control of bradyzoite specific *LDH2* promoter so that the brain cysts containing bradyzoites can readily be identified by fluorescence microscopy [18]. The deletion of the *CST1* (*Δcst1 T. gondii* strain) was verified by PCR (Figure S1B) as well as by RNA-seq (Figure S1C).

*T. gondii* lysates of parasites grown *in vitro* at pH 8.1 (bradyzoites) and pH 7 (tachyzoites) were probed with mAb SalmonE by immunoblot (Figure 4B). The mAb SalmonE reactive band at pH 8.1 is seen in the stacking gel, suggesting that this is a high molecular mass antigen with extensive post translational glycosylation that may prevent entry into the resolving gel. While the mAb SalmonE reactivity is virtually absent in parasites grown at pH 7, a strong signal was observed in lysates from wild-type parasites grown at pH 8.1. These CST1 bands also bind to DBA lectin consistent with the presence of glycosylation in this protein (Figure S2).

**Figure 5:**
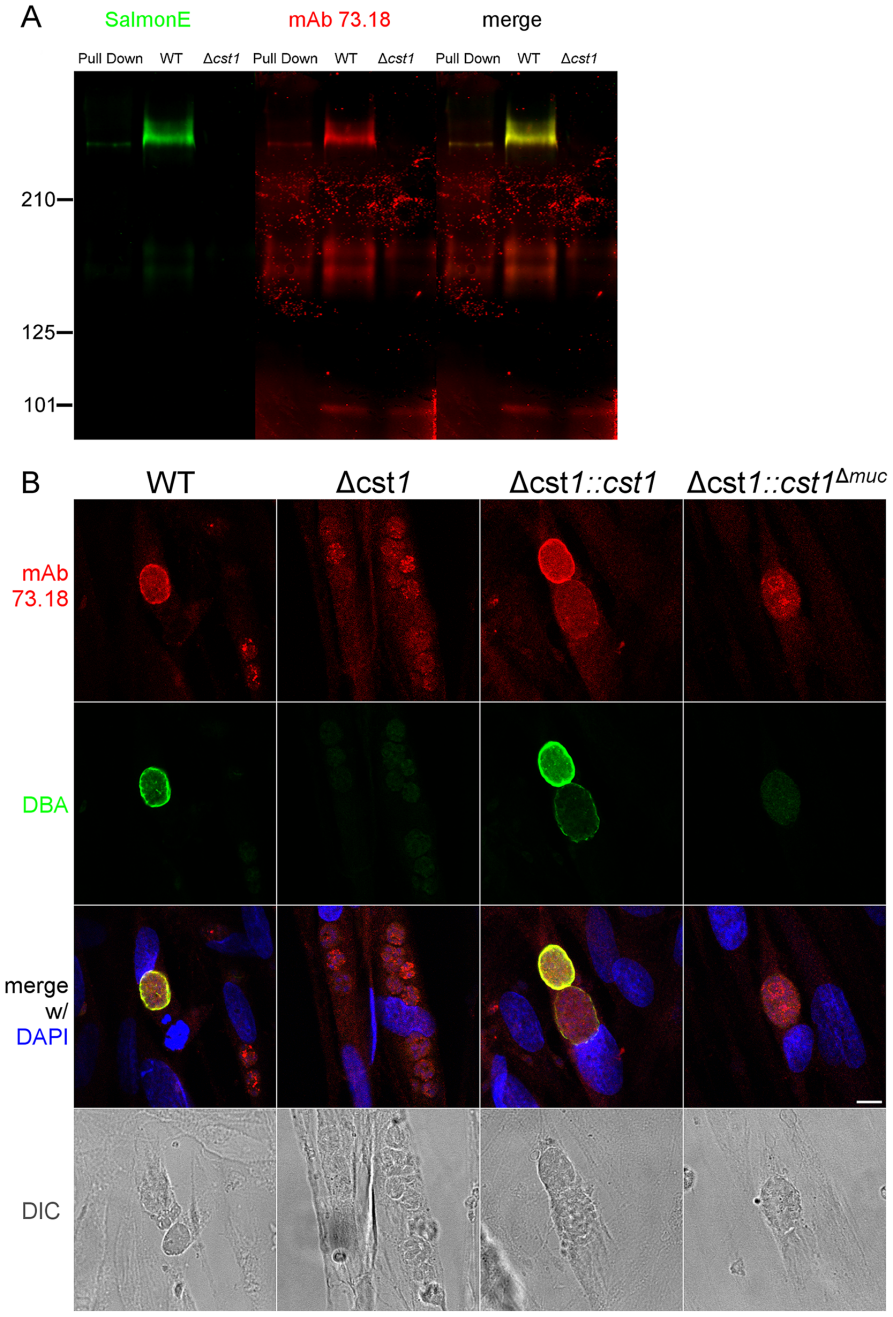
SalmonE and mAb 73.18 recognize CST1 (TGME49_064660). (**A**) Immunoblot of mAb SalmonE immunoprecipitated material (ME49), WT lysate (Pru) and Δ*cst1* lysate probed with mAb SalmonE (green) and mAb 73.18 (red). This immunoblot demonstrated that mAb SalmonE and mAb 73.18 bind to the same major species which is not detectable in Δ*cst1* parasites. (**B**) IFA of *in vitro* cysts probed with CST1 specific monoclonal antibody 73.18 (red) and DBA lectin (green). The presence of full-length CST1 is required for both mAb 73.18 and DBA cyst wall staining. Bar, 10 µm.

**Figure 6:**
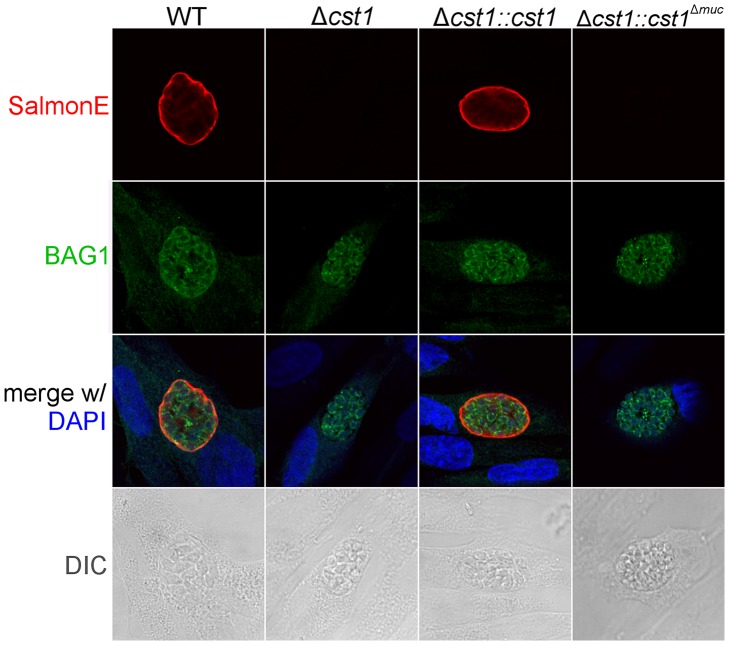
BAG1 expression in WT, Δ*cst1*, Δ*cst1::cst* and Δ*cst1::cst1* ^Δ^ ^***muc***^ **parasites.** HFF cells were infected with either WT, Δ*cst1*, Δ*cst1::cst* and Δ*cst1::cst1* ^Δ*muc*^ parasites and probed with anti-CST1 antiserum (red) and rabbit anti-BAG1 (green). This demonstrates that differentiation occurs in the Δ*cst1* and Δ*cst1::cst1* ^Δ*muc*^ parasites.

**Figure 7:**
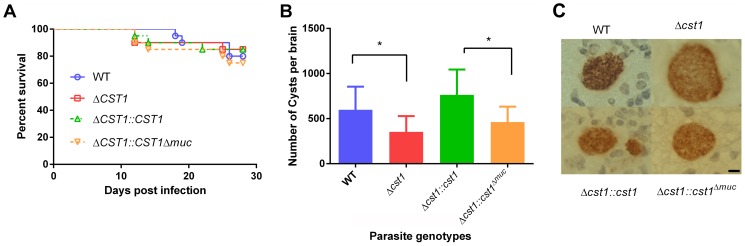
Brain cyst burden and survival rate of infected mice at 4 weeks post infection. (**A**) Survival curve of the mice challenged with the WT and mutant parasites (n = 20). (**B**) Brain cyst count of C57/BL6 mice infected with either WT, Δ*cst1*, Δ*cst1:cst1* or Δ*cst1:cst1* ^Δ*muc*^ parasites sacrificed at 4 weeks post infection. The bars represent mean and standard deviation (n = 12). *p<0.05 (Mann-Whitney U test). (**C**) Immunohistochemistry of brain sections from infected mice probed with anti-GFP antibody demonstrates the formation of brain cysts *in vivo*. Pru parasites are stably transfected with *LDH2-GFP* enabling identification of bradyzoites within cysts. Bar, 10 µm.

Since CST1 was originally defined by reactivity to DBA and recognition by mAb 73.18 [10], [11], the CST1 deficient strain (*Δcst1*) should not be recognized by mAb 73.18. Immunopurified CST1, and cell lysates from pH 8.1 treated *T. gondii* Pru wild type or *Δcst1* cultures were probed with the previously described CST1 specific mAb 73.18 and mAb SalmonE (Figure 5A). Monoclonal antibody SalmonE and mAb 73.18 had similar patterns of reactivity on immunoblot, and, as expected, the *Δcst1* did not have the major immunoreactive band and had lost the characteristic cyst wall labeling seen with DBA, mAb SalmonE (Figure 4C), or mAb 73.18 (Figure 5B). These cysts that did not stain with DBA, mAb SalmonE or 73.18 were still positive with BAG1 antibody (Figure 6). Examination of brains of mice infected with *Δcst1 T. gondii* demonstrated that cyst formation could still occur in this knockout strain (Fig. 7).

Collectively, these results indicate CST1 is not required for bradyzoite or cyst formation and that CST1 is the cyst wall protein recognized by mAb SalmonE, DBA, and mAb 73.18. Both DBA and mAb 73.18 recognize glycoepitopes, and we noted that while cyst wall reactivity was lost in *Δcst1*, there was some residual reactivity seen within the parasites by mAb SalmonE, DBA and mAb 73.18, suggesting that other less abundant glycoepitopes that react with mAb SalmonE, DBA, and mAb 73.18 are present in bradyzoites.

### Complementation of *Δcst1* strain reveals that the mucin domain is required for DBA lectin reactivity

To examine the role of glycosylation in CST1 function, we complemented the Δ*cst1* strain with two variants of CST1 (Figure 4A). One Δ*cst1* line was complemented with a full-length cDNA (Δ*cst1::cst1*) and the other with a CST1 lacking the 789 bp region coding for the mucin domain (Δ*cst1::cst1* ^Δ*muc*^). Following transfection and selection, the presence of those complemented genes were verified by PCR (Figure S1B) and RNA-seq (Figure S1C). Stage specific expression of CST1 was equivalent to that seen in the wild type parasite in the complemented Δ*cst1::cst1 T. gondii* strain as verified by immunoblot with mAb SalmonE (Figure 4B). IFA of pH 8.1 treated Δ*cst1::cst1 in vitro* cysts demonstrated that the strain complemented with full-length cDNA of CST1 has the correct localization of CST1 to the cyst wall, as well as the restoration of DBA staining (Figure 4C) and mAb 73.18 staining (Figure 5B) of the cyst wall. These cysts remained IFA positive for BAG1 (Figure 6).

In contrast, the Δ*cst1::cst1* ^Δ*muc*^ parasites lack the major mAb SalmonE and DBA reactive band in immunoblot (Figure 4B and S2), and lack cyst wall staining with DBA, mAb SalmonE, and mAb 73.18 in IFA (Figure 4C and 5B). The expression and localization of CST1^Δmuc^ protein was verified with a polyclonal antibody produced against recombinant CST1 (rTGME49_064660 AA1-200) (Figure S3).

### Δ*cst1* parasites form fewer brain cysts and exhibit a fragile cyst wall phenotype

To determine the effect of CST1 deletion or mucin domain deletion *in vivo*, C57BL/6 mice were infected with wild type, Δ*cst1*, Δ*cst1::cst1*, and Δ*cst1::cst1* ^Δ*muc*^ parasites at 200 parasites per mouse. The mouse survival rates (Figure 7A) during acute infection with each parasite line were not statistically different (n = 20, Log-rank test). The number of brain cysts per mouse at 4 weeks after infection (Figure 7B) was reduced by 41% in the Δ*cst1* strain (p<0.05, Mann-Whitney U test). Complementation with full length (Δ*cst1::cst1*), but not mucin-null (Δ*cst1::cst1* ^Δ*muc*^), restored the cyst number level back to the wild type levels. Histological analysis of the brains suggested that inflammation was less severe in Δ *cst1* than the wild type (Figure S4); however this did not achieve statistical significance. Cysts were produced *in vivo* by all mutants (Figure 7C) and there was no difference in the size of cysts produced by these mutants. Brains from mice infected with the wild type, Δ *cst1*, Δ *cst1::cst1* ^Δ*muc*^, and Δ*cst1::cst1* parasites were fed to Balb/c^DM1^ and all were capable of transmitting infection.

**Figure 8:**
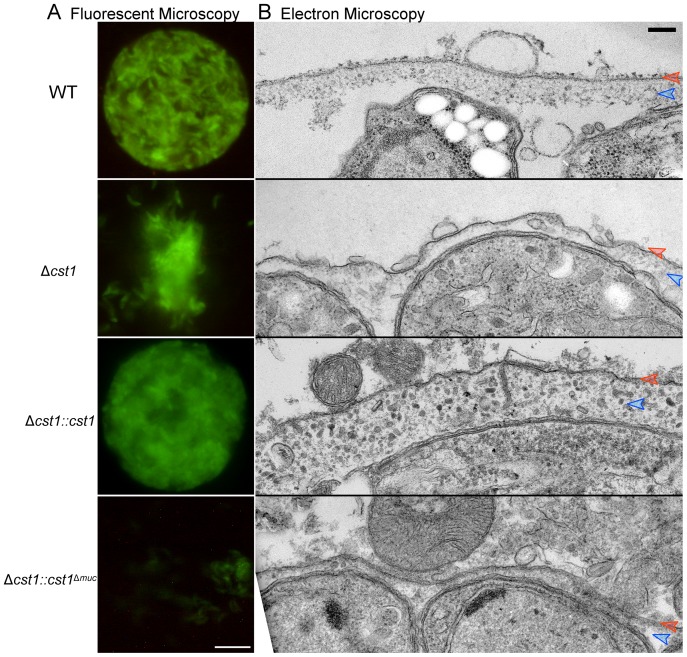
The mucin domain of CST1 is required to form mechanical stress-resistant cyst wall structures. (**A**) Brain cysts of WT and mutants after gentle brain homogenization, observed under epifluorescence microscopy. Bar, 10 µm. (**B**) Electron micrograph of *T. gondii* brain cysts demonstrates an alteration in the cyst wall with disruption and loss of the granular layer found underneath the cyst wall membrane. This disruption is visible in the Δ*cst1* and Δ*cst1::cst1* ^Δ*muc*^ cysts, but no disruption is seen in the WT and Δ*cst1::cst1* cysts. Red arrows indicate the cyst wall membrane and the blue arrows indicate the cyst wall granular layer. Measurements of the cyst wall: WT 153±28 nm, Δ*cst1* 24±10 nm, Δ*cst1::cst1* 284±65 nm, and Δ *cst1::cst1* ^Δ*muc*^ 34±14 nm (p<0.05 WT vs Δ*cst1*, WT vs Δ *cst1::cst1* ^Δ*muc*^, and WT vs Δ *cst1::cst1*). The original image magnification was 20,000× on all of these EM images. Bar, 200 nm.

During the brain cyst isolation procedure, cysts are subjected to mechanical stress to release them from brain tissue to purify them by isopycnic centrifugation [19]. The wild type cysts stayed intact during this procedure, but Δ*cst1* brain cysts were much more fragile and broke apart when homogenized using a pestle tissue homogenizer to purify cysts from brain samples [19] (Figure 8A). Despite many attempts, we were unable to develop a reliable procedure to purify intact Δ*cst1* cysts from mouse brains or a method to standardize cyst inocula to compare the transmissibility of cysts from our mutant strains.

To further investigate the fragile phenotype, we examined the ultrastructure of the brain cysts by electron microscopy. Figure 8B demonstrates a wild type brain cyst, which has the classic organized cyst wall with an underlying amorphous granular layer. In contrast, the Δ*cst1* brain cysts lack this organization and displayed a disrupted layer. Independently isolated Δ*cst1* clones had the same fragile phenotype. Full length cDNA complementation of Δ*cst1* (Δ*cst1::cst1*) rescued the fragile brain cyst phenotype as well as restoring the cyst wall layer as seen by TEM (Figure 8). In contrast, complementation of Δ*cst1* parasite with *cst1* ^Δ*muc*^ gene did not rescue the fragile cyst wall phenotype or correct the disruption of cyst wall layer seen by TEM (Figure 8). Measurements of the cyst wall confirmed a significant decrease in the cyst wall thickness with disruption of cst1: WT 153±28 nm, Δ*cst1* 24±10 nm, Δ*cst1::cst1* 284±65 nm, and Δ *cst1::cst1* ^Δ*muc*^, 34±14 nm (p<0.05 WT vs Δ*cst1*, WT vs Δ *cst1::cst1* ^Δ*muc*^) as well as an increase in cyst wall thickness in the Δ*cst1::cst1* strain (p<0.05 WT vs Δ *cst1::cst1*).

To determine if cyst wall thickness affected cyst fragility, we compared cyst fragility in individual brains using a relatively vigorous disruption method with a small sintered glass pestle tissue homogenizer (size A: 0.1–0.15 mm clearance, frosted inner glass surface) that disrupts a significant fraction of wild-type cysts in brain homogenate. For this experiment, brains (n = 4 per group) were cut in half, the cysts in the right half were homogenized unfixed, and the left half was fixed with 4% paraformaldehyde overnight at 4°C prior to homogenization with the pestle tissue homogenizer. Using this procedure 100±0% of *Δcst1* and 100±0% *Δcst1::cst1^Δmuc^* cysts were broken. Interestingly, fewer *Δcst1::cst1* (15±3%) cysts were broken than wild type cysts (61±8%), suggesting that the increased thickness of the cyst wall seen on TEM (Figure 8) with *Δcst1::cst1* parasites does protect the cysts/bradyzoites from mechanical stress.

### Δ*cst1* parasites replicate slower during *in vitro* bradyzoite development

**Figure 9:**
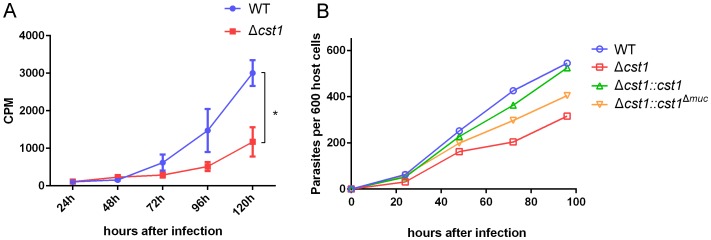
Growth of Δ*cst1* parasite is impaired at pH 8.1 but not at pH 7.1. (**A**) Growth of WT (blue) or Δ*cst1* parasites (red) in HFF cells at pH 8.1 were measured as ^3^H-uracil incorporation into parasites DNA. Mean and standard deviation are shown. n = 3, * *p*<0.005. This experiment was repeated 3 times with similar results. (**B**) Number of parasites inside vacuoles in HFF cells at pH 8.1 per 600 host cells. ^3^H-uracil incorporation was not done due to the lack of UPRT gene in complement strains.

To examine any growth defects of the Δ*cst1* parasite, parasite growth was measured with incorporation of ^3^H-uracil in pH 7.1 (tachyzoite stage) and pH 8.1 (bradyzoite differentiation) medium. The deletion of CST1 resulted in a reduction in the growth rate at pH 8.1 of the Δ*cst1* parasite compared to the growth rate of the wild type (WT) parasites (Figure 9A, *p*<0.005), this reduction in growth was not seen at pH 7 (Figure S5). This slower growth phenotype seen with bradyzoite inducing condition in Δ*cst1* parasites was rescued by full length CST1 complementation, but only partially by the mucin-null CST1 (Figure 9B).

### The Δ*cst1* strain has dysregulated expression of bradyzoite and pH-induced genes

**Figure 10:**
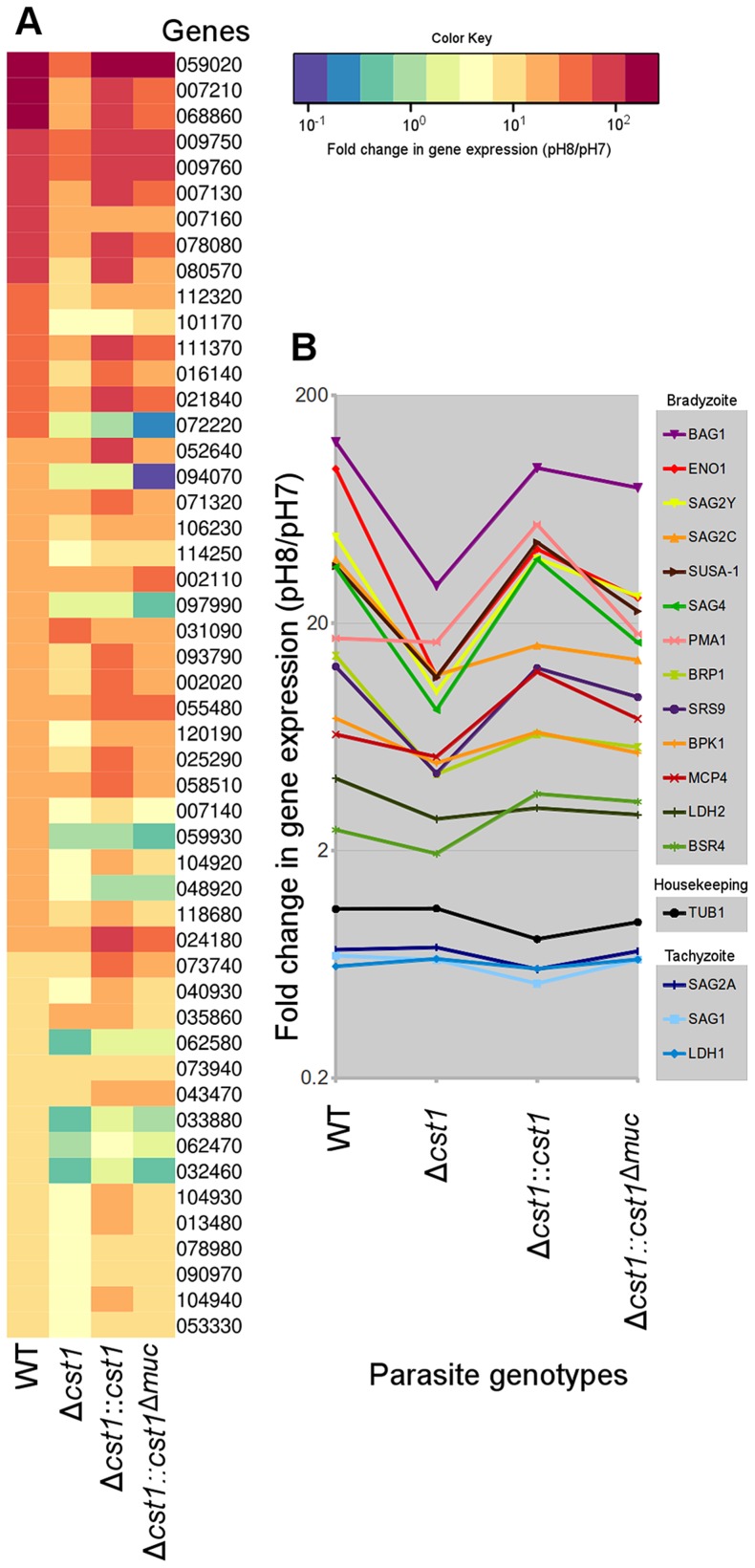
Lack of CST1 reduces bradyzoite gene expression. (**A**) Heat map of fold change in gene expression (pH 8/pH 7) of wild type and cst1 mutants. The top 50 upregulated genes in wild type *T. gondii* are displayed in order. The number is the gene name in TGME49 (ToxoDB ver 6.1). Gene upregulation is reduced in the Δ*cst1* strain. The full length complement strain Δ*cst1:cst1* has a restoration of gene upregulation to control (WT) levels, but the mucin-null complement strain Δ*cst1:cst1* ^Δ*muc*^ did not have restoration of these gene levels. (**B**) Upregulation of known bradyzoite specific gene expressions shows the same pattern. Note that the housekeeping and tachyzoite specific genes did not follow the same expression pattern. Both (A) and (B) graphs were generated from the same RNA-seq data set.

Since there was reduction in growth rate at pH 8.1 *in vitro* for the Δ*cst1* parasite and fewer brain cysts *in vivo*, we investigated whether the *cst1* mutants might have global changes in gene expression under the bradyzoite-inducing conditions. The transcriptome of parasites cultured at pH 7 (tachyzoite) was compared with the transcriptome of parasites cultured at pH 8.1 (bradyzoite) for 3 days using RNA-seq. Figure 10A shows the heat map of top 50 upregulated genes at bradyzoite conditions in wild type (WT) *T. gondii* (see Table S1 for a complete list of these genes). Expression of 49 of 50 genes was less efficiently induced by pH shock in the Δ*cst1* strain compared to control WT parasites. The complementation of full-length CST1 (Δ*cst1::cst1*) restored the majority of these genes back to their wild type level. However, the mucin-null complement (Δ*cst1::cst1* ^Δ*muc*^) was not able to restore expression of these genes to wild type levels. Figure 10B shows the fold change in gene expression for several known bradyzoite specific genes. These bradyzoite genes also follow a similar pattern of reduced gene upregulation with Δ*cst1*, restoration with Δ*cst1::cst1* and only a partial restoration with Δ*cst1::cst1* ^Δ*muc*^. The altered gene expression pattern was not evident for housekeeping or tachyzoite specific genes. This RNA-seq data suggests that lack of CST1 disrupts bradyzoite differentiation, but not enough to prevent *in vitro* and *in vivo* cyst formation.

## Discussion

This study identifies the gene encoding the major cyst wall DBA-binding protein CST1. CST1 is a SRS containing protein with an extended mucin domain. The mucin domain of CST1 is necessary for DBA binding and is a major domain for glycosylation of this protein. Δ*cst1* parasites can differentiate and form mouse brain cysts without CST1; however, CST1 and CST1 glycosylation is required for formation of an organized cyst wall layer that confers structural rigidity to the cyst wall. This is the first cyst wall protein that has been shown to be essential to establish the physical integrity of *in vivo* brain cysts. Complementation demonstrates that the mucin domain of CST1 is necessary for the cyst wall organization and rigidity. In addition to its role in structural stability of the cyst wall, a lack of CST1 also reduces *in vitro* growth rate, mouse brain cyst number, and pH 8-induced bradyzoite specific gene upregulation in *T. gondii*. These results suggest that expression of CST1 or glycosylation of CST1 in early cyst development influences the expression pattern of genes during bradyzoite differentiation.

Previous work has suggested that the cyst wall contains several glycoproteins including CST1 [10], a proteophosphoglycan [11] and other unknown glycoproteins reacting with s-WGA [8]. CST1 is highly unusual SRS protein in that it has a large mucin domain and thirteen SRS domains. The glycoprotein gp900, from another Apicomplexan parasite *Cryptosporidium parvum*, has a large mucin-like domain that has 68% sequence similarity with the mucin domain of CST1. Other than the presence of mucin-like domain, there is no sequence similarity between these two glycoproteins. The *C. parvum* gp900 has a transmembrane domain, is expressed on the plasma membrane, and is shed into the environment [20]. The gp900 protein is localized to the tethers on the inner surface of oocyst walls [20], [21]. This suggests that gp900, a large mucin-like protein, is important for making a structurally rigid enclosure for this parasite. Other smaller glycoproteins (gp40 and gp15) are present in the oocyst wall tethers of *C. parvum* [20], [21]. Our studies with CST1 in *T. gondii* tissue cysts suggest phylogenetic conservation of the functions of these secreted structural glycoproteins in the Apicomplexa.

The protozoan parasite *Trypanosoma cruzi* has up to 850 highly glycosylated and GPI-anchored surface mucin genes that form a stage specific mosaic coat on their cell surface. It is suggested that these glycoproteins have a protective role against the proteases in the intestine of the insect vector [22], function in attachment to the host cell, and as an immune evasion mechanism [23]. CST1, which has large mucin domain and a predicted GPI-anchor (as with other SRS domain containing proteins), may have comparable functions in terms of protecting bradyzoites in the cyst from the proteases present in the gastrointestinal tract during the oral infection or in the surrounding necrotic tissues when the host dies [22]. The ability of mucins to retain large amount of water probably protects parasites by preventing dehydration, facilitating parasite transmission to the next host.

Of the 189 cyst-wall positive hybridomas we identified, 34 had an identical immunoblot pattern as mAb SalmonE. This suggests that CST1 may be highly immunogenic toward the Th2 pathway. Cysts are present not only in the central nervous system, but also in the visceral organs and muscles. CST1 from ruptured cysts can induce a strong antibody response and this may facilitate the clearance of cysts by the immune system when their host cells are dead. Cloned hybridomas specific to CST1 included other classes of antibodies (e.g. IgG2b and IgM), therefore, CST1 does not only elicit the production of IgE antibody.

In a previous study, CST1 was detected by its reactivity to the mAb 73.18 in the R5 strain of *T. gondii*, an atovoquone resistant mutant that spontaneously formed cysts more readily [24]. In this strain CST1 was reported as a 116 kDa band on 2D SDS-PAGE [10]. In our current study, both mAb SalmonE and mAb 73.18 detected two distinct bands (Figures 5A, S6), one migrating at 150 kDa (SRS13, Weiss, unpublished data) and one band (CST1) in the stacking gel (>210 kDa). Similar immunoblot patterns were seen using Pru, ME49 or the ME49 mutant R5 (Figure S6). Both bands also react with DBA lectin indicating that the SRS13 has similar glycoepitope as CST1. This SRS13 reactivity is still present in the Δ*cst1* strain and does not localize to cyst wall (Tomita and Weiss, unpublished data) and mAb SalmonE staining of the 150 kDa band is absent in a Δ*srs13* strain (Figure S6). Monoclonal antibody SalmonE, mAb 73.18, and DBA all display no cyst wall staining in the Δ*cst1* strain, consistent with SRS44 being CST1, a major cyst wall glycoprotein identified by our laboratory over 15 years ago [10], [11]. During previous studies we were not able to detect the band in the stacking gel since the stacking gel was separated from the resolving gel before the transfer to a membrane. The discrepancy in the molecular-weight of 116 kDa and 150 kDa may be due to the difference in gel conditions or molecular markers.

CST1 is one of several cyst wall proteins that are induced during bradyzoite development. Recently, a screening of insertional mutants for a reduction of *in vitro* cyst development led to the identification of another *T. gondii* cyst wall protein, proteophosphoglycan (TgPPG) [25]. TgPPG is expressed in the cyst wall and is probably highly glycosylated, as evidenced by its retention in the stacking gel on SDS-PAGE. Disruption of TgPPG gene results in the delay in cyst wall formation and bradyzoite conversion; however, complementation only rescued cyst wall formation measured by the DBA staining, but not bradyzoite differentiation as measured by BAG1 expression. In another recent study, transcriptomic analysis of brain cysts yielded two distinct cyst wall proteins Bradyzoite Pseudokinase 1 (BPK1) and Microneme Adhesive Repeat domain-containing protein 4 (MCP4) [13]. Subsequent study demonstrated that BPK1 plays a role in effective oral transmission [26]. Finally, several GRA proteins have been demonstrated to localize the cyst wall in bradyzoite parasitophorous vacuoles, as well as to the dense granules in both tachyzoites and bradyzoties [27]. Deletion of cyst wall associated GRA6 was shown to dramatically decrease tissue cyst burdens in mice [18]. The biological functions of these cyst wall proteins await further study.

*Toxoplasma gondii* is one of the most successful parasites partly because it forms persistent latent cysts that last for the life of its hosts, and the cyst wall is a critical biological structure for this persistence. CST1 functions as a key structural component reinforcing the cyst wall structure and conferring resistance to physical stress to the *T. gondii* cyst. The fragile cyst phenotype in the Δ*cst1* strain suggests that transmission and persistence could be affected by this gene deletion, but this hypothesis could not be fully tested, as intact viable cysts cannot be purified from mouse brains to perform quantitative oral challenge experiments [10], [11].

There are 5 putative UDP:GalNAc:polypeptide N-acetylgalactosaminyltransferases (ppGalNAc-T) in the *T. gondii* genome (Toxo DB). Two of these, T1 and T3, were expressed in insect cells and their glycosyl transferase activity was confirmed [28]. Both transferases turned out to be “follow-up” transferases, only transfering to the pre-O-glycosylated peptide. At this point, it is not known which glycosyl transferasase is responsible for the heavy glycosylation of CST1 mucin domain. A recent study showed that the nucleotide sugar transporter (TgNST1) is necessary for the glycosylation of cyst wall proteins such as CST1 [29]. Deletion of *TgNST1* resulted in fewer brain cysts and the authors concluded that glycosylation of the cyst wall is required for persistence of bradyzoites, but did not identify which glycoproteins were most critical. Our studies show a similar phenotype for parasites lacking CST1, consistent with CST1 being the major cyst wall glycoprotein involved in the maintenance of brain cyst burden. Furthermore, parasites expressing a mutant CST1 lacking the mucin domain have a similar defect, demonstrating that post translational glycosylation of the CST1 mucin domain is critical for CST1 biological function. Therefore enzymes involved in glycosylation of cyst wall proteins including candidate enzymes such as *T. gondii* ppGalNAc-Ts have potential as therapeutic agents to prevent *T. gondii* bradyzoite persistence.

## Materials and Methods

### *T.gondii* cell culture and strains

Human foreskin fibroblasts (HFF) were maintained in 10% fetal bovine serum pH 7 DMEM with penicillin-streptomycin at 5% CO_2_. Confluent monolayers were infected with Type II strains ME49, the reference genome strain, or Pru*Δku80* strain of *T. gondii*, which is widely used for genetic studies [18]. For *in vitro* bradyzoite differentiation, parasite strains were grown in differentiation medium (DMEM medium adjusted to pH 8.1 with 10 mM HEPES and 1% fetal bovine serum with penicillin-streptomycin) for 3 days at 0.5% CO_2_.

### Ethics statement

All animal experiments were conducted according to the U.S.A. Public Health Service Policy on Humane Care and Use of Laboratory Animals. Animals were maintained in an AAALAC-approved facility and all protocols were approved by the Institutional Care Committee of the Albert Einstein College of Medicine, Bronx, New York(Animal Protocols 20121104, 20121109 and 20121110; Animal Welfare Assurance number A3312-01).

No human samples were used in these experiments. Human foreskin fibroblasts were obtained from ATCC.

### Monoclonal antibody production

BALB/c^dm1^ mice, which have a deletion in the *Ld* gene at the *HLA-2L* locus and produce more brain cysts than wild type BALB/c [30], were infected with ME49 strain of *T. gondii* and treated with sulfamerazine at 30 mg/L in drinking water to minimize death from the acute infection. Four weeks after the infection, brain cysts were isolated using previously described isopycnic centrifugation [19]. Briefly, brains were isolated and homogenized in PBS with a pestle tissue homogenizer with clearance of 0.15–0.23 mm (Thomas Scientific) for 10 times. Percoll was added to 40% of the total volume and centrifuged at 27,000×g for 20 minutes. The middle layer was recovered and centrifuged with equal volume of PBS at 100×g for 10 minutes. Cysts were then subjected to ten freeze-thaw cycles, (3 minutes each: 100% ethanol-dry ice bath followed by room temperature water bath) and emulsified with an equal volume of Freund's complete adjuvant. The emulsion was injected into BALB/c mice subcutaneously. Two months later, spleens were isolated from the immunized mice and fused with myeloma cell line to create hybridoma libraries. Using the IFA, ELISA and immunoblot, those hybridoma supernatants were screened against parasites that were cultured in pH 8.1 medium. IFA patterns were similar for two Type II strains ME49 and Pru. Subcloned hybridoma cells were cultured in CELLine bioreactor (Integra) for large-scale production of monoclonal antibodies.

### Transmission electron microscopy

The BALB/c^dm1^ mice were infected with Pru strain of *T. gondii* for 4 weeks in the presence of sulfamerazine at 30 mg/L in drinking water. After they were harvested, whole brains were fixed in 4% paraformaldehyde in PBS for overnight, followed by homogenization and isopycnic centrifugation as described above. While wild-type cysts did not require fixation prior to processing, fixation of Δ*cst1* infected brains was necessary in order to prevent breakage of fragile brain cysts. The isolated cysts were fixed with 2.5% glutaraldehyde, 2% paraformaldehyde in 0.1M sodium cacodylate buffer, postfixed with 1% osmium tetroxide followed by 2% uranyl acetate, dehydrated through a graded series of ethanol and embedded in LX112 resin (LADD Research Industries, Burlington VT). Ultrathin sections were cut on a Reichert Ultracut UCT, stained with uranyl acetate followed by lead citrate and viewed on a JOEL 1200EX transmission electron microscope at 80 kv.

For immunoelectron microscopy, the cysts were fixed with 4% paraformaldehyde 0.05% glutaraldehyde in 0.1M sodium cacodylate buffer, dehydrated through a graded series of ethanol, with a progressive lowering of the temperature to −50°C in a Leica EMAFS, embedded in Lowicryl HM-20 monostep resin (Electron Microscopy Sciences), and polymerized using UV light. Ultrathin sections were cut on a Reichert Ultracut E, immunolabeled with SalmonE, and then stained with uranyl acetate followed by lead citrate. Stained sections were viewed on a JOEL 1200EX transmission electron microscope at 80 kv.

### Identification of the SalmonE reactive glycoprotein by mass spectrometry

SalmonE was crosslinked to the Protein L agarose beads with disuccinimidyl suberate following the manufacturer's protocol (Thermo Scientific). Human foreskin fibroblasts were infected with ME49 strain of *T. gondii* and incubated for 3 days in pH 8.1 medium with 10% FBS at 0.5% CO_2_. Cells were lysed with 1% TritonX-100 in PBS with proteinase inhibitor cocktail and incubated with SalmonE-beads for 2 hours at 4°C. The beads were extensively washed with 1% TritonX-100 PBS and eluted with 0.1M glycine at pH 2.5. The eluate was neutralized and separated on SDS-PAGE. The gel was stained with Coomassie Brilliant Blue and a visible high molecular-weight band was excised. The protein in the band was reduced and alkylated using TCEP and iodoacetamide then digested with trypsin in 25 mM ammonium bicarbonate/0.01% ProteaseMax at 50°C for 1 hour. The resulting digest was cleaned with C18 ziptip and the peptides eluted onto a MALDI plate with a saturated solution of α-cyanohydroxycinnamic acid in 70% acetonitrile/0.1% trifluoroacetic acid. MS/MS analysis of the digested sample was carried out using the AB Sciex 4800 MALDI-TOF-TOF (Applied Biosystems), operated at 20 kV accelerating voltage in the reflector positive ion mode. The MS/MS data generated were converted to mgf files and searched against EPICDB [31] using the in-house Mascot Protein Search engine (Matrix Science) for protein identification.

### Production of CST1 mouse antisera

RNA was isolated from ME49 strain of *T. gondii* and a cDNA library was created using SuperScript III First Strand Kit (Invitrogen). The first 200 peptides of the CST1 (TGME49_064660), as predicted in ToxoDB (i.e. this does not include the probable N-terminal extension of *CST1* gene predicted using SignalP 4.0 [15]), was amplified by PCR, cloned into the pET32 vector and used to transform BL21 competent *E. coli*. Recombinant CST1 protein was expressed using Overnight Express Autoinduction System 1 (Novagen), purified with nickel column, and separated on SDS-PAGE. The band was cut out and emulsified with Freund's complete adjuvant and immunized into BALB/c mice intraperitoneally. Three months after the immunization, antisera were collected and probed against *in vitro* cysts using IFA.

### CST1 (TGME49_064660; SRS44) knock-out

Type II Prugniaud strain with deletion of the *Ku80 gene* (Pru*Δku80*) [18] was used as the background strain for the creation of Δ*cst1* strain. The construct for the knockout was built as previously described [18]. Briefly, 1 kb upstream and downstream genomic DNA sequence of TGME_064660 gene were amplified from the parental Pru*Δku80* strain. These fragments were concatenated into pRS416 yeast shuttle vector (ATCC) flanking the selectable marker hypoxanthine-xanthine-guanine phosphoribosyltransferase [*HXGPRT*] cassette using the yeast strain ATCC#90845. This construct deletes whole CST1 gene as well as 205 nucleotides 5′ upstream region from the predicted start site. This 5′ region includes the probable start site and signal peptides. All the primers used in the plasmid construct are listed in the Supplemental material (Table S2). The parasites were transfected with the linearized Δ*cst1* vector and subcloned in the presence of 25 µg/ml mycophenolic acid and 50 µg/ml xanthine. Integration of Δ*cst1* vector at the TGME49_064660 locus was verified by PCR. Lack of CST1 protein expression was confirmed with IFA and immunoblot.

### Complementation of *CST1*

For *CST1* complementation, DNA fragments were concatenated into pSMART-BAC plasmid (Lucigen) using In-Fusion system (Clontech) following the manufacture's protocol. The homologous sequences 1 kb upstream and downstream of UPRT coding region for the UPRT locus were isolated from genomic DNA. Flanking sequences 1.3 kb 5′ and 3′ of *CST1* were isolated from Pru genomic DNA. TGME49_064660 cDNA was generated from RNA harvested from Pru strain cultured at pH 8.1. The fragments were concatenated in the following order, 5′ UPRT recombination sequence, TGME49_064660 upstream element, TGME49_064660 cDNA, TGME49_064660 3′ UTR, and 3′ UPRT recombination sequence.

For the mucin domain null mutant complementation, two fragments (base 1–6006 and 6766–7035 with appropriate adapters) were used instead of whole cDNA. This mucin domain null vector replaces the mucin domain (nt 6007–6765) with a 1xFLAG sequence. The parasites were transfected with complementation vectors and subcloned in the presence of 5 µM 5-fluorodeoxyuridine (FUDR). Integration of complementing vectors at the *UPRT* locus was verified with PCR.

Our attempts to use the *HXGPRT* selectable marker present in the Δ*cst1* parasite to complement the endogenous *cst1* locus were not successful. This was probably due to the low level expression of *HXGPRT* at the *cst1* locus that was not sufficient for negative selection using 6-thioxanthine, but was sufficient for positive selection in constructing the knockout using mycophenolic acid (MPA). We have experienced similar problems using the Δ*ku80* system with the HGXPRT selectable marker and have found that removal of this marker by negative selection is not feasible at all loci and needs to be evaluated for each knock-out. Therefore complementation was performed at the *UPRT* locus for the *cst1* gene since this target provides a direct selection and the loss of UPRT does not influence cyst development or cyst burdens in mice [18].

### 5′ RACE

RNA was isolated using RNeasy mini with DNase treatment from HFF infected with ME49 strain of *T. gondii* culture in pH 8.1 medium for 3 days. 5′ UTR was amplified with FirstChoice RLM-RACE kit (Ambion) with gene specific primer GGGCGGGTCGAAAATGTTG following the manufacturer's protocol. The DNA was sequenced with ABI 3730 DNA analyzer.

### Immunofluorescence assay

Cells were fixed with 4% paraformaldehyde in PBS for 30 minutes on ice and then permeablized with 0.2% TritonX100/0.1% glycine/0.2% bovine serum albumin (BSA) in PBS on ice for 20 minutes. The cells were washed with 0.2% BSA in PBS three times and blocked with 1% BSA in PBS at 4°C overnight. All incubation with primary and secondary antibodies was done at 37°C for 90 minutes in a moist chamber. Concentration of the antibodies and probes used were 20 µg/ml for FITC-conjugated DBA (Vector Lab), 1∶50 for antibody 73.18, 1∶100 for SalmonE, 1∶25 for mouse polyclonal anti-CST1(1–200), 1∶50 for rabbit anti-BAG, 1∶200 anti-FLAG M2 (Sigma) all in 1% BSA/PBS. Cells were washed three times with 0.2% BSA/PBS then incubated with appropriate secondary antibody at 1∶500. After incubation, the cells were washed three times with 0.2% BSA/PBS and mounted with ProLong Gold antifade (Invitrogen). Photomicrographs were taken either with a SP5 confocal microscope (Leica) or Microphoto-FXA epifluorescence microscope (Nikon).

### Immunoblotting

HFF cells were infected with *T. gondii* in normal DMEM medium or differentiation medium and kept in 5% or 0.5% CO_2_ respectively. Cells were harvested at 3 dpi with cell scraper, centrifuged at 3000×g for 15 minutes and lysed with 1% TritonX100, 1% SDS, protease inhibitor cocktail in PBS. The samples were resolved in 10% SDS-PAGE at 100 V and transferred at 250 mA for 90 minutes on the PVDF membrane Immobilon-FL (Millipore). The membrane was blocked overnight with 5% non-fat dry milk (NFDM) in PBST, then probed with SalmonE at 1∶250 and anti-GRA1 at 1∶500 in 1% BSA/PBS at 4°C on shaker overnight. Following antibody incubation the membrane was washed three times with PBST and then incubated with donkey anti-mouse antibody conjugated with IRDye800 (Licor) at 1∶40,000 in 5% NFDM/PBST at room temperature for 90 minutes. The membrane was then scanned using an Odyssey (Licor) imaging system.

For the Immunoprecipitation-Immunoblot experiments, SDS was omitted from the lysis buffer to minimize the disruption of antigen-antibody interaction. Immunoprecipitation was performed as described in the mass spectrometry method section. The gel was transferred to nitrocellulose membrane and probed with DBA conjugated with alkaline phosphatase (EY Laboratories) at 1∶100 in PBST for 90 minutes at room temperature. The membrane was then washed three times with PBST and developed with BCIP/NBT color development substrate (Promega). The image of the membrane was scanned using a desktop scanner. For double labeling with SalmonE and 73.18 (CST1 reactive; [10], [11], samples were run in 6% SDS-PAGE gel with 3% stacking gel. The membrane was probed with 73.18 (1∶20) then with anti-mouse IRDye700 (1∶20000). After the membrane was scanned, it was re-probed with SalmonE (1∶100) then rat anti-mouse IgE antibody (1∶2000) then anti-rat IRDye800 (1∶20000).

### Parasite growth assay

For WT and Δ*cst1* parasites, a growth assay was performed using a previously described ^3^H-uracil incorporation method [32]–[34]. Since mammalian host cells are not capable of uracil uptake but *T. gondii* is [33], [34], the incorporation of radio-labeled uracil is used to measure the growth of parasites. Briefly, HFF monolayers in 12-well tissue culture plates were infected (100 or 10,000 parasites/well) in either pH 7.1 or pH 8.1 medium and incubated in 5% or 0.5% CO_2_ respectively. Cells were incubated with 1 ml of 2 µCi/ml ^3^H-uracil per well for 24 hours before the each harvest time point. At 24, 48, 72, or 96 hours after infection, medium was removed and cells were lysed with 1% SDS and 100 µg/ml of unlabeled uracil in PBS on ice. Nucleic acids were precipitated with 10% trichloroacetic acid on ice for 2 hours. The contents of each well were filtered with the glass fiber filters. The radioactivity of ^3^H on the filter was measured with a scintillation counter.

For assessment of Δ*cst1::cst1* and Δ*cst1::cst1* ^Δ*muc*^ parasite growth, microscopic observation of the growth of intracellular parasites was used, as the disruption of the UPRT gene locus results in parasites that no longer incorporate uracil. Parasites were grown as described above then fixed in 4% paraformaldehyde, permeabilized with 0.2% TritonX100 and probed with anti-GRA1 antibody to visualize the parasites. For pH 8 (bradyzoite permissive condition) a total of 600 host cells were examined for each time point/condition and the number of intracellular parasites per 600 cells determined. Bradyzoite vacuoles have been reported to be resistant to lysis so direct counting of vacuoles was used to evaluate growth. For pH 7 (tachyzoite permissive condition) parasites were grown as described above (10,000 parasite initial inoculum), cells were scraped from each well of a 24 well plate, centrifuged and then re-suspended in 100 µl of 0.5% saponin/PBS, pipetted 20 times to ensure host cell lysis and then counted in a Neubauer hemocytometer in triplicate.

### Parasite challenge, cyst count, and histology

Female 4 to 8 week old C57/BL6 mice (Jackson Laboratory) were infected with 200 tachyzoites intraperitoneally. Any observed mortality was recorded until 28 days after infection when the mice were sacrificed and brains harvested. Brains were fixed in 4% PFA in PBS overnight. Right brain halves were partially homogenized with a syringe with PBS into 600 µl suspension then 120 µl were counted under fluorescent microscope. Left halves of each brain were fixed in 10% neutral buffered formalin for additional 72 hours and processed for paraffin embedding. Samples for histopathology were sectioned to a thickness of 5 µm and stained using hematoxylin and eosin (H&E) stains. Slides were analyzed for the presence of tissue cysts, inflammation, and gliosis by light microscopy and graded on a scale of 0–5 (where 0 = no lesions; 1 = minimal lesions; 2 = mild lesions; 3 = moderate lesions; 4 = marked lesions; and 5 = severe lesions). Sections were graded by the pathologist in a blinded fashion to avoid confirmation bias.

### Deep RNA sequencing (RNA-seq)

HFF cells grown in seven 150 mm tissue culture plates were infected with parasites for each strain WT Pru, Δ*cst1*, Δ*cst1*::*cst1* and Δ*cst1*::*cst1* ^Δ*muc*^ in regular medium (pH 7 DMEM with 10% fetal bovine serum, incubated in 5% CO_2_). Eight hours later, free parasites were removed by washing with PBS and replaced with regular medium (pH 7, 5% CO_2_) or differentiation medium (pH 8.1 DMEM with 1% fetal bovine serum, 10 mM HEPES, incubated in 0.5% CO_2_). Three days after the infection, cells were harvested, passed through 27G needle twice to lyse HFF cells and filtered through 3 µm pore polycarbonate membrane to remove HFF cells. This separation process was performed on ice. Purified parasites were pelleted at 1000×g for 15 minutes 4°C.

RNA was extracted using TRIzol Reagent (Invitrogen), followed by genomic DNA removal and cleaning using RNase-Free DNase Set kit and Mini RNease kit (Qiagen). Integrity of the RNA samples was assessed using the Aligent 2100 Bioanalyzer. RNA samples having RNA Integrity Number between 9 and 10 were used in this work. MicroPoly(A)Purist Kit (Ambion) was used for enrichment of transcripts. The SOLiD Total RNA-Seq Kit was used to construct template cDNA for RNA-Seq following the protocol recommended by Applied Biosystems. Briefly, mRNA was fragmented using chemical hydrolysis followed by ligation with strand specific adapters and reverse transcription was used to generate cDNA. The cDNA fragments, 150 to 250 bp in size, were subsequently isolated by electrophoresis in 6% Urea-TBE acrylamide gel. The isolated cDNA was amplified through 15 amplification cycles to produce the required number of templates for the SOLiD EZ Bead system, which was used to generate the template bead library for ligation base sequencing by the either SOLiD4 or 5500xl SOLiD instrument (LifeTechnologies). The 50-base short read sequences produced by the SOLiD sequencer were mapped in color space using the Whole Transcriptome analysis pipeline in Life Technologies LifeScope software version 2.5 against the genome of *T. gondii* strain ME49 using the default mapping setting. Both Fasta and GFF files were obtained from ToxoDB website (www.toxoDB.org; Release 6.1). The output of the Whole Transcriptome analysis generated (1) a gene counts file, with the base counts summed to a single value across the entire gene length, and with a RPKM value also given for each gene; (2) a BAM file containing the sequence of every mapped read and its mapped location; (3) two pairs of *.wig files (one pair for the two strands on each chromosome) giving the mapped counts at each base position; and (4) a statistics summary on alignment and filtering report. Fold change in gene upregulation at bradyzoite induction was calculated by dividing the RPKM values of pH 8.1 by that of pH 7. The genes with low level expression (RPKM<5) in pH 8.1 WT parasites were removed. The top 50 genes that were upregulated in WT parasites were plotted with gplot heatmap.2 in R.

## Supporting Information

Figure S1 **(A) Schematic of 5′ locus for the TGME49_064660 (cst1) gene.** This figure shows the 5′ region of TGME49_064660 (cst1) locus. The blue region is the 5′ UTR that was experimentally determined using 5′ RACE. The brown arrows indicate the predicted start site (from www.ToxoDB.org), which does not contain the signal sequence, and the putative upstream start site, which includes the predicted signal sequence. The number indicates the number of nucleotides from the end of upstream gene TGME49_064670. **(B) Presence of CST1 genes in parasites.** PCR was performed (upper panel) with genomic *T. gondii* DNA using primers flanking an intron and the mucin domain in order to show the presence of the genes. The amplicon size expected from each version of *cst1* gene is: gDNA 1824 bp, cDNA 1546 bp, cDNA^Δ*muc*^ 781 bp. A second PCR was performed (lower panel) with primers upstream of *cst1* and in the center of the selectable marker *HXGPRT* to demonstrate the insertion of the Δ*cst1* vector into the *cst1* locus (1321 bp). **(C) RNA sequencing data demonstrating the expression of CST1 genes.** Sequence reads of pH shocked parasite RNA mapped to TGME49 genome (ToxoDB) at the *cst1* locus. Type I strain (RH) and Type II (ME49) both express *cst1* mRNA. The *cst* knockout (Δ*cst1*) does not express *CST1*, whereas the wild type (WT), cDNA complement (Δ*cst1*::*cst1*) and mucin null complement (Δ*cst1*::*cst1^Δmuc^*) all express CST1.
(PDF)

Figure S2 **SalmonE and DBA.** (**A**) Parasite total lysate (same membrane as Figure 2) was probed with DBA lectin. Both mAb SalmonE (green) and DBA lectin (red) react with the same high molecular band (CST1). Lower green bands (at the 25 kDa) are GRA1 used as a parasite loading control. (**B**) mAb SalmonE Densitometry. Densitometry measurements for each band and the normalized CST1 expression level (CST/GRA1) for each lane are shown in the following table and figure.
(PDF)

Figure S3 **CST1Δmuc protein is expressed and localized to the cyst wall.** HFF cells were infected with either WT or Δ*cst1::cst1* ^Δ*muc*^ parasites and probed with anti-CST1 antiserum (red) and DBA (green). This demonstrates that the CST1^Δmuc^ protein is expressed and localized to the cyst wall, that DBA lectin binding is lost in *CST1* ^Δ*muc*^ parasites.
(PDF)

Figure S4 **Histology of infected murine brains.** (**A**) Hematoxylin and eosin (H&E) stained brain sections were scored for meningoencephalomyelitis using a scale of 0 to 5 (n = 4). p<0.05 WT vs Δ*cst1::cst1* ^Δ*muc*^. (**B**) Brains from mice infected with WT, Δ*cst1*, Δ*cst1*::*cst1*, or Δ*cst1*::*cst1^Δmuc^* parasites for 4 weeks were sectioned and stained with H&E. Photomicrographs were obtained at 4×, 10× and 20× magnifications. Tissue cysts are indicated by a red arrow on 20× magnification images.
(PDF)

Figure S5 **Parasite growth measured at pH 7.0.** **A.** Growth measurement using ^3^H Uracil. Growth of WT (blue) or Δ*cst1* parasites (red) in HFF cells (10,000 parasites per well at time zero) at pH 7 was measured as ^3^H-uracil incorporation into parasite DNA. Mean and standard deviation are shown. n = 3. This experiment was repeated 3 times and had similar results for wells harvested in triplicate. **B.** Growth measurement by counting parasites. Growth of WT (blue), Δ*cst1* (red), Δ*cst1*::*cst1* (green), Δ*cst1*::*cst1^Δmuc^* (yellow) parasites at pH 7 culture condition was measured by lysing the host HFF cells with 0.5% saponin and counting the free parasites in a hemocytometer.
(PDF)

Figure S6 **Immunoblot using SalmonE and 73.18 to R5, ME49 and Pru strains of** ***T. gondii*** **and to Δ** ***cst1*** **and Δ** ***srs13*** **strains in Pru** ***Δku80 T. gondii*** **.** This demonstrates that mAb SalmonE binds to several type II strain *T. gondii* parasites and that a similar sized band is seen in these parasites. It also demonstrates that monoclonal 73.18 binds to this high molecular weight band (recognized by SalmonE) as well as a smaller band that we have identified as SRS13 (Tomita and Weiss unpublished).
(PDF)

Table S1 **List of top 50 upregulated genes in bradyzoites.** Gene up-regulation was determined by the RNA-seq RPKM value at pH 8 divided by that at pH 7. The genes are listed in order of highest upregulation. Gene ID and corresponding descriptions are from ToxoDB.
(PDF)

Table S2 **Primers used for construction of plasmids for the deletion and complementation of** ***CST1*** **.**
(PDF)

## References

[1] Tenter AM, Heckeroth AR, Weiss LM (2000) *Toxoplasma gondii*: from animals to humans. International Journal for Parasitology 30: 1217–1258. 11113252 10.1016/s0020-7519(00)00124-7 PMC3109627

[2] Pappas G, Roussos N, Falagas ME (2009) Toxoplasmosis snapshots: global status of *Toxoplasma gondii* seroprevalence and implications for pregnancy and congenital toxoplasmosis. International Journal for Parasitology 39: 1385–1394. 19433092 10.1016/j.ijpara.2009.04.003

[3] Ferguson DJ, Hutchison WM (1987) The host-parasite relationship of *Toxoplasma gondii* in the brains of chronically infected mice. Virchows Archiv A, Pathological anatomy and histopathology 411: 39–43. 3107207 10.1007/BF00734512

[4] Hill D, Dubey JP (2002) Toxoplasma gondii: transmission, diagnosis and prevention. Clinical Microbiology and Infection 8: 634–640. 12390281 10.1046/j.1469-0691.2002.00485.x

[5] Ferguson DJ, Hutchison WM, Pettersen E (1989) Tissue cyst rupture in mice chronically infected with *Toxoplasma gondii*. An immunocytochemical and ultrastructural study. Parasitology Research 75: 599–603. 2771928 10.1007/BF00930955

[6] Dubey JP, Lindsay DS, Speer CA (1998) Structures of Toxoplasma gondii Tachyzoites, Bradyzoites, and Sporozoites and Biology and Development of Tissue Cysts Clinical Microbiology Reviews. 11: 267–299. 10.1128/cmr.11.2.267 PMC106833 9564564

[7] Sims TA, Hay J, Talbot IC (1988) Host-parasite relationship in the brains of mice with congenital toxoplasmosis. The Journal of Pathology 156: 255–261. 3204455 10.1002/path.1711560311

[8] Boothroyd JC, Black M, Bonnefoy S, Hehl A, Knoll LJ, et al (1997) Genetic and biochemical analysis of development in *Toxoplasma gondii* . Philosophical transactions of the Royal Society of London Series B, Biological sciences 352: 1347–1354. 9355126 10.1098/rstb.1997.0119 PMC1692023

[9] Weiss LM, Kim K (2000) The development and biology of bradyzoites of *Toxoplasma gondii* . Frontiers in Bioscience 5: D391–405. 10762601 10.2741/weiss PMC3109641

[10] Zhang YW, Halonen SK, Ma YF, Wittner M, Weiss LM (2001) Initial characterization of CST1, a Toxoplasma gondii cyst wall glycoprotein. Infection and Immunity 69: 501–507. 11119543 10.1128/IAI.69.1.501-507.2001 PMC97909

[11] Weiss LM, LaPlace D, Tanowitz HB, Wittner M (1992) Identification of *Toxoplasma gondii* bradyzoite-specific monoclonal antibodies. The Journal of Infectious Diseases 166: 213–215. 1376757 10.1093/infdis/166.1.213

[12] Weiss LM, Kim K (2007) Bradyzoite development. In: Weiss LM, Kim K, editors. Toxoplasma gondii: The Model Apicomplexan. Elsevier Ltd. pp. 341–366.

[13] Buchholz KR, Fritz HM, Chen X, Durbin-Johnson B, Rocke DM, et al (2011) Identification of tissue cyst wall components by transcriptome analysis of in vivo and in vitro *Toxoplasma gondii* bradyzoites. Eukaryotic Cell 10: 1637–1647. 22021236 10.1128/EC.05182-11 PMC3232729

[14] Wasmuth JD, Pszenny V, Haile S, Jansen EM, Gast AT, et al (2012) Integrated Bioinformatic and Targeted Deletion Analyses of the SRS Gene Superfamily Identify SRS29C as a Negative Regulator of Toxoplasma Virulence. mBio 3: e00321–12. 23149485 10.1128/mBio.00321-12 PMC3509429

[15] Petersen TN, Brunak S, Von Heijne G, Nielsen H (2011) SignalP 4.0: discriminating signal peptides from transmembrane regions. Nature Methods 8: 785–786. 21959131 10.1038/nmeth.1701

[16] Julenius K, Gupta R (2005) Prediction, conservation analysis, and structural characterization of mammalian mucin-type O-glycosylation sites. Glycobiology 15: 153–164. 15385431 10.1093/glycob/cwh151

[17] Etzler ME, Kabat EA (1970) Purification and characterization of a lectin (plant hemagglutinin) with blood group A specificity from *Dolichos biflorus* . Biochemistry 9: 869–877. 4984730 10.1021/bi00806a022

[18] Fox BA, Falla A, Rommereim LM, Tomita T, Gigley JP, et al (2011) Type II *Toxoplasma gondii* KU80 knockout strains enable functional analysis of genes required for cyst development and latent infection. Eukaryotic Cell 10: 1193–1206. 21531875 10.1128/EC.00297-10 PMC3187049

[19] Cornelissen AWCA, Overdulve JP, Hoenderboom JM (1981) Separation of *Isospora (Toxoplasma) gondii* cysts and cystozoites from mouse brain tissue by continuous density - gradient centrifugation. Parasitology 83: 103. 6267543 10.1017/s0031182000050071

[20] Barnes DA, Bonnin A, Huang JX, Gousset L, Wu J, et al (1998) A novel multi-domain mucin-like glycoprotein of *Cryptosporidium parvum* mediates invasion. Molecular and Biochemical Parasitology 96: 93–110. 9851610 10.1016/s0166-6851(98)00119-4

[21] Chatterjee A, Banerjee S, Steffen M, O'Connor RM, Ward HD, et al (2010) Evidence for mucin-like glycoproteins that tether sporozoites of *Cryptosporidium parvum* to the inner surface of the oocyst wall. Eukaryotic Cell 9: 84–96. 19949049 10.1128/EC.00288-09 PMC2805294

[22] Mortara Ra, Da Silva S, Araguth MF, Blanco Sa, Yoshida N (1992) Polymorphism of the 35- and 50-kilodalton surface glycoconjugates of *Trypanosoma cruzi* metacyclic trypomastigotes. Infection and Immunity 60: 4673–4678. 1328061 10.1128/iai.60.11.4673-4678.1992 PMC258217

[23] Buscaglia Ca, Campo Va, Frasch ACC, Di Noia JM (2006) *Trypanosoma cruzi* surface mucins: host-dependent coat diversity. Nature Reviews Microbiology 4: 229–236. 16489349 10.1038/nrmicro1351

[24] McFadden DC, Tomavo S, Berry EA, Boothroyd JC (2000) Characterization of cytochrome b from *Toxoplasma gondii* and Qo domain mutations as a mechanism of atovaquone-resistance. Molecular and Biochemical Parasitology 108: 1–12. 10802314 10.1016/s0166-6851(00)00184-5

[25] Craver MPJ, Rooney PJ, Knoll LJ (2010) Isolation of *Toxoplasma gondii* development mutants identifies a potential proteophosphogylcan that enhances cyst wall formation. Molecular and Biochemical Parasitology 169: 120–123. 19879901 10.1016/j.molbiopara.2009.10.006 PMC2791180

[26] Buchholz KR, Bowyer PW, Boothroyd JC (2013) Bradyzoite Pseudokinase 1 Is Crucial for Efficient Oral Infectivity of the *Toxoplasma gondii* Tissue Cyst. Eukaryotic Cell 12: 399–410. 23291621 10.1128/EC.00343-12 PMC3629768

[27] Ferguson DJJP (2004) Use of molecular and ultrastructural markers to evaluate stage conversion of *Toxoplasma gondii* in both the intermediate and definitive host. International Journal for Parasitology 34: 347–360. 15003495 10.1016/j.ijpara.2003.11.024

[28] Stwora-Wojczyk MM, Dzierszinski F, Roos DS, Spitalnik SL, Wojczyk BS (2004) Functional characterization of a novel *Toxoplasma gondii* glycosyltransferase: UDP-N-acetyl-D-galactosamine:polypeptide N-acetylgalactosaminyltransferase-T3. Archives of Biochemistry and Biophysics 426: 231–240. 15158673 10.1016/j.abb.2004.02.013

[29] Caffaro CE, Koshy Aa, Liu L, Zeiner GM, Hirschberg CB, et al (2013) A Nucleotide Sugar Transporter Involved in Glycosylation of the Toxoplasma Tissue Cyst Wall Is Required for Efficient Persistence of Bradyzoites. PLoS Pathogens 9: e1003331. 23658519 10.1371/journal.ppat.1003331 PMC3642066

[30] Brown C (1990) Class I MHC genes and CD8+ T cells determine cyst number in *Toxoplasma gondii* infection. The Journal of Immunology 145: 3438–3441. 2121825

[31] Madrid-Aliste CJ, Dybas JM, Angeletti RH, Weiss LM, Kim K, et al (2009) EPIC-DB: a proteomics database for studying Apicomplexan organisms. BMC Genomics 10: 38. 19159464 10.1186/1471-2164-10-38 PMC2652494

[32] Zhang YW, Kim K, Ma YF, Wittner M, Tanowitz HB, et al (1999) Disruption of the *Toxoplasma gondii* bradyzoite-specific gene BAG1 decreases in vivo cyst formation. Molecular Microbiology 31: 691–701. 10027984 10.1046/j.1365-2958.1999.01210.x PMC3109652

[33] Pfefferkorn ER, Pfefferkorn LC (1977) *Toxoplasma gondii*: specific labeling of nucleic acids of intracellular parasites in Lesch-Nyhan cells. Experimental Parasitology 41: 95–104. 851481 10.1016/0014-4894(77)90134-5

[34] Schwartzman JD, Pfefferkorn ER (1981) Pyrimidine synthesis by intracellular *Toxoplasma gondii* . The Journal of Parasitology 67: 150–158. 7241272

