# Supplementary material for: The Toxoplasma gondii Cyst Wall Protein CST1 Is Critical for Cyst Wall Integrity and Promotes Bradyzoite Persistence
Source: PLoS Pathog. 2013 Dec 26;9(12):e1003823. doi: 10.1371/journal.ppat.1003823 (PMC3873430; doi:10.1371/journal.ppat.1003823)

Figure S1. (A) Schematic of 5' locus for the TGME49_064660 (cst1) gene. This figure shows the 5' region of TGME49_064660 (cst1) locus. The blue region is the 5' UTR that was experimentally determined using 5' RACE. The brown arrows indicate the predicted start site (from www.toxodb.org), which does not contain the signal sequence, and the putative upstream start site, which includes the predicted signal sequence. The number indicates the number of nucleotides from the end of upstream gene TGME49_064670.

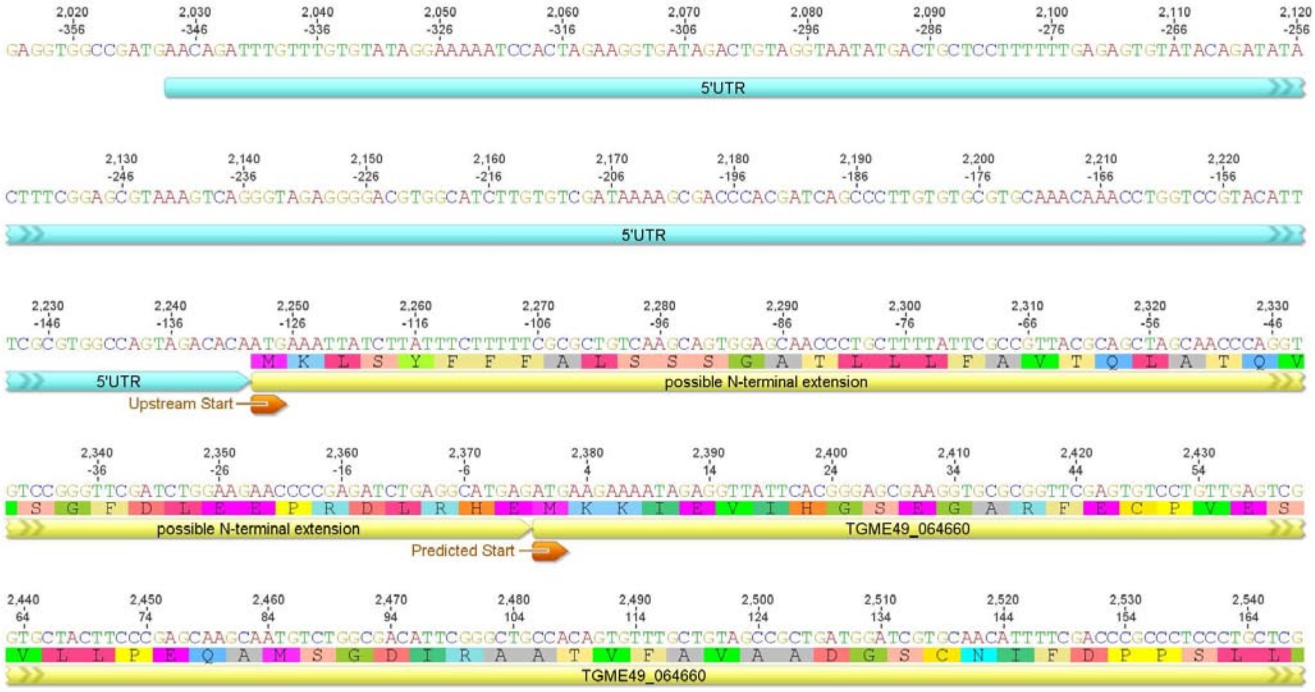

Figure S1. (B) Presence of CST1 genes in parasites. PCR was performed (upper panel) with genomic *T. gondii* DNA using primers flanking an intron and the mucin domain in order to show the presence of the genes. The amplicon size expected from each version of *cst1* gene is: gDNA 1824 bp, cDNA 1546 bp, cDNA$^{\Delta muc}$ 781 bp. A second PCR was performed (lower panel) with primers upstream of *cst1* and in the center of the selectable marker *HXGPRT* to demonstrate the insertion of the Δ*cst1* vector into the *cst1* locus (1321 bp).

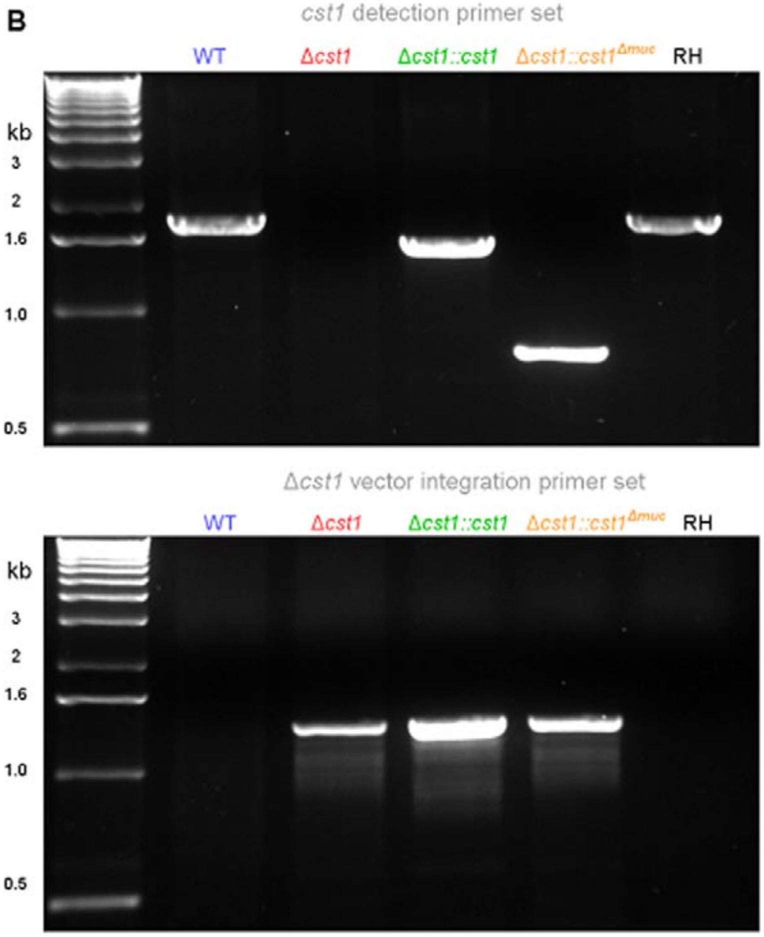

Figure S1. (C) RNA sequencing data demonstrating the expression of CST1 genes. Sequence reads of pH shocked parasite RNA mapped to TGME49 genome (ToxoDB) at the *cst1* locus. Type I strain (RH) and Type II (ME49) both express *cst1* mRNA. The *cst* knockout (Δ*cst1*) does not express *CST1*, whereas the wild type (WT), cDNA complement (Δ*cst1*::*cst1*) and mucin null complement (Δ*cst1*::*cst1*$^{\Delta muc}$) all express CST1.

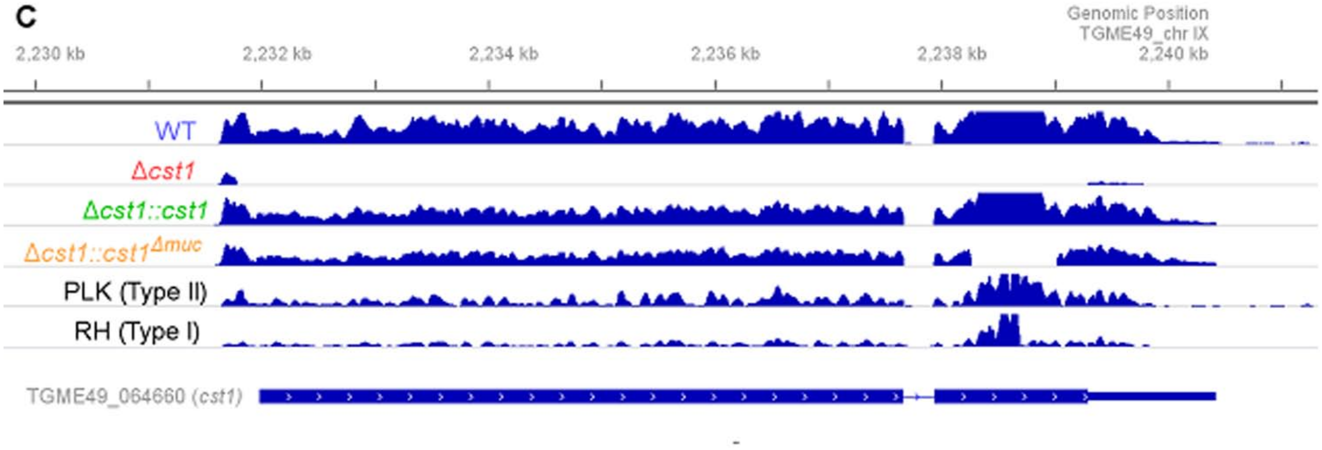

Supplement: Figure S1 — (A) Schematic of 5′ locus for the TGME49_064660 (cst1) gene. This figure shows the 5′ region of TGME49_064660 (cst1) locus. The blue region is the 5′ UTR that was experimentally determined using 5′ RACE. The brown arrows indicate the predicted start site (from www.ToxoDB.org), which does not contain the signal sequence, and the putative upstream start site, which includes the predicted signal sequence. The number indicates the number of nucleotides from the end of upstream gene TGME49_064670. (B) Presence of CST1 genes in parasites. PCR was performed (upper panel) with genomic T. gondii DNA using primers flanking an intron and the mucin domain in order to show the presence of the genes. The amplicon size expected from each version of cst1 gene is: gDNA 1824 bp, cDNA 1546 bp, cDNAΔmuc 781 bp. A second PCR was performed (lower panel) with primers upstream of cst1 and in the center of the selectable marker HXGPRT to demonstrate the insertion of the Δcst1 vector into the cst1 locus (1321 bp). (C) RNA sequencing data demonstrating the expression of CST1 genes. Sequence reads of pH shocked parasite RNA mapped to TGME49 genome (ToxoDB) at the cst1 locus. Type I strain (RH) and Type II (ME49) both express cst1 mRNA. The cst knockout (Δcst1) does not express CST1, whereas the wild type (WT), cDNA complement (Δcst1::cst1) and mucin null complement (Δcst1::cst1Δmuc) all express CST1. (PDF) [file ppat.1003823.s001.pdf]
