# Supplementary material for: The Toxoplasma gondii Cyst Wall Protein CST1 Is Critical for Cyst Wall Integrity and Promotes Bradyzoite Persistence
Source: PLoS Pathog. 2013 Dec 26;9(12):e1003823. doi: 10.1371/journal.ppat.1003823 (PMC3873430; doi:10.1371/journal.ppat.1003823)
Supplement: Figure S2 — SalmonE and DBA. (A) Parasite total lysate (same membrane as Figure 2) was probed with DBA lectin. Both mAb SalmonE (green) and DBA lectin (red) react with the same high molecular band (CST1). Lower green bands (at the 25 kDa) are GRA1 used as a parasite loading control. (B) mAb SalmonE Densitometry. Densitometry measurements for each band and the normalized CST1 expression level (CST/GRA1) for each lane are shown in the following table and figure. (PDF) [file ppat.1003823.s002.pdf]

Figure S2. (A) mAb SalmonE and DBA

Parasite total lysate (same membrane as Figure 4B) was probed with DBA lectin. Both mAb SalmonE (green) and DBA lectin (red) reacts with the same high molecular band (CST1). Lower green bands (at the 25 kDa marker) are GRA1 used as a parasite loading control.

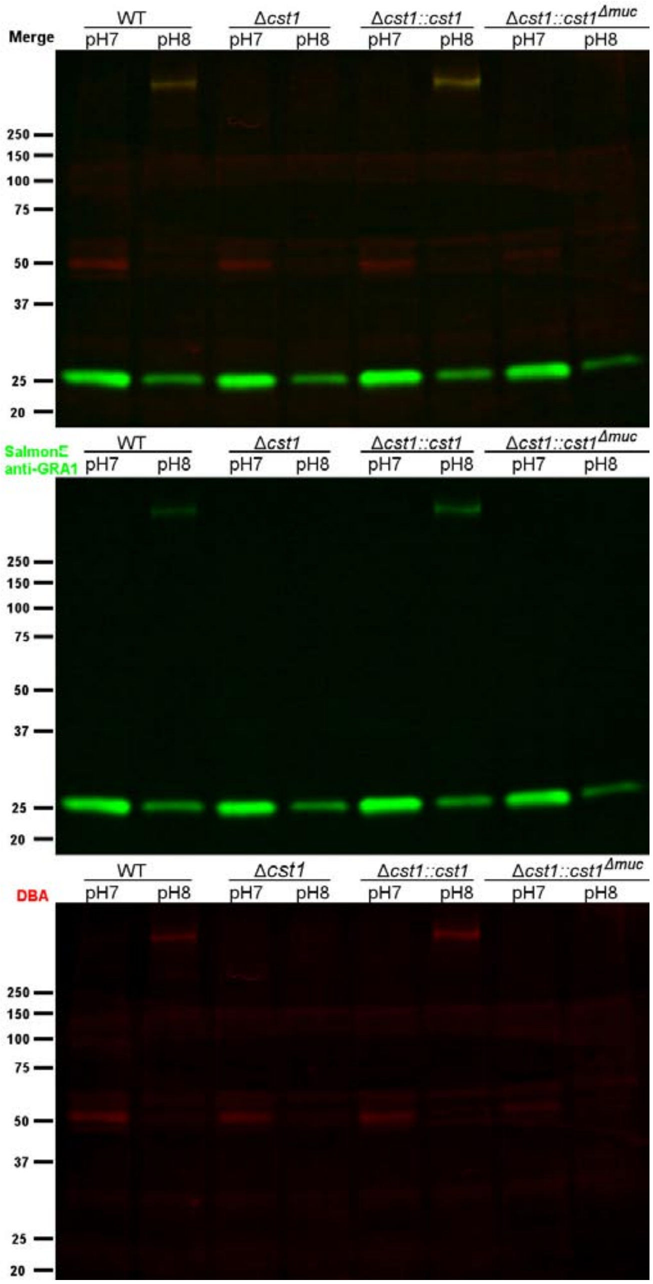

FIGURE S2 (B) mAb SalmonE Densitometry

Densitometry measurements for each band and the normalized CST1 expression level (CST/GRA1) for each lane are shown in the following table and figure

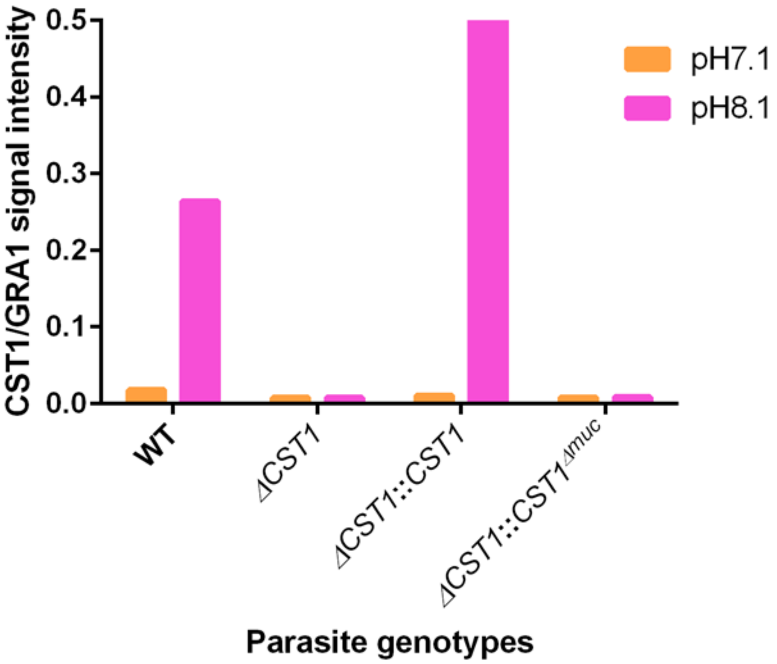

	WT pH7	WT pH8	ΔCST1 pH7	ΔCST1 pH8	ΔCST1::CST1 pH7	ΔCST1::CST1 pH8	ΔCST1::CST1Δmuc pH7	ΔCST1::CST1Δmuc pH8
**CST1/GRA1**	**0.018**	**0.264**	**0.008**	**0.008**	**0.011**	**0.503**	**0.008**	**0.009**
**CST1**	**21400**	**102000**	**8520**	**2770**	**14100**	**194000**	**9110**	**2300**
**GRA**	**1180000**	**386000**	**1070000**	**343000**	**1270000**	**386000**	**1080000**	**259000**
