# Supplementary material for: The Toxoplasma gondii Cyst Wall Protein CST1 Is Critical for Cyst Wall Integrity and Promotes Bradyzoite Persistence
Source: PLoS Pathog. 2013 Dec 26;9(12):e1003823. doi: 10.1371/journal.ppat.1003823 (PMC3873430; doi:10.1371/journal.ppat.1003823)

Figure S3. CST1Δmuc protein is expressed and localized to the cyst wall.

HFF cells were infected with either WT or Δ*cst1::cst1*$^{\Delta muc}$ parasites and probed with anti-CST1 antiserum (red) and DBA (green). This demonstrates that the CSTΔmuc protein is expressed and localized to the cyst wall, that DBA lectin binding is lost in *CST1*$^{\Delta muc}$ parasites.

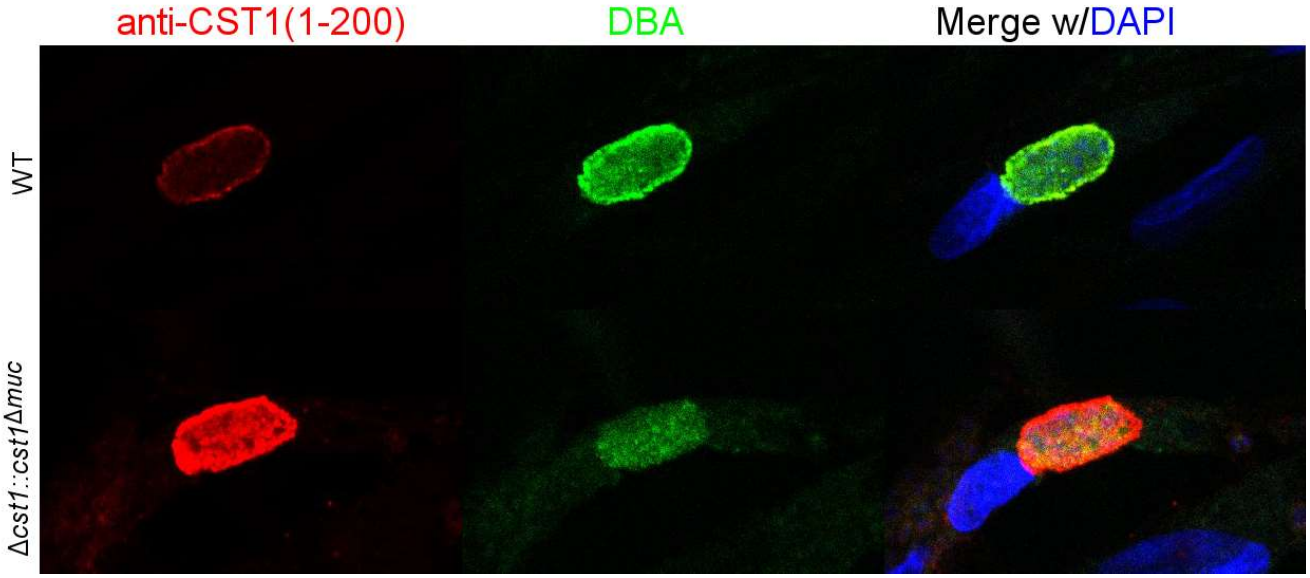

Supplement: Figure S3 — CST1Δmuc protein is expressed and localized to the cyst wall. HFF cells were infected with either WT or Δcst1::cst1 Δmuc parasites and probed with anti-CST1 antiserum (red) and DBA (green). This demonstrates that the CST1Δmuc protein is expressed and localized to the cyst wall, that DBA lectin binding is lost in CST1 Δmuc parasites. (PDF) [file ppat.1003823.s003.pdf]
