# Supplementary material for: The Toxoplasma gondii Cyst Wall Protein CST1 Is Critical for Cyst Wall Integrity and Promotes Bradyzoite Persistence
Source: PLoS Pathog. 2013 Dec 26;9(12):e1003823. doi: 10.1371/journal.ppat.1003823 (PMC3873430; doi:10.1371/journal.ppat.1003823)

Figure S4. Histology of infected murine brains

Figure S4 (A)

(A) Hematoxylin and eosin (H&E) stained brain sections were scored for meningoencephalomyelitis using a scale of 0 to 5 (n=4). $p<0.05$ WT vs Δ*cst1::cst1*$^{\Delta muc}$

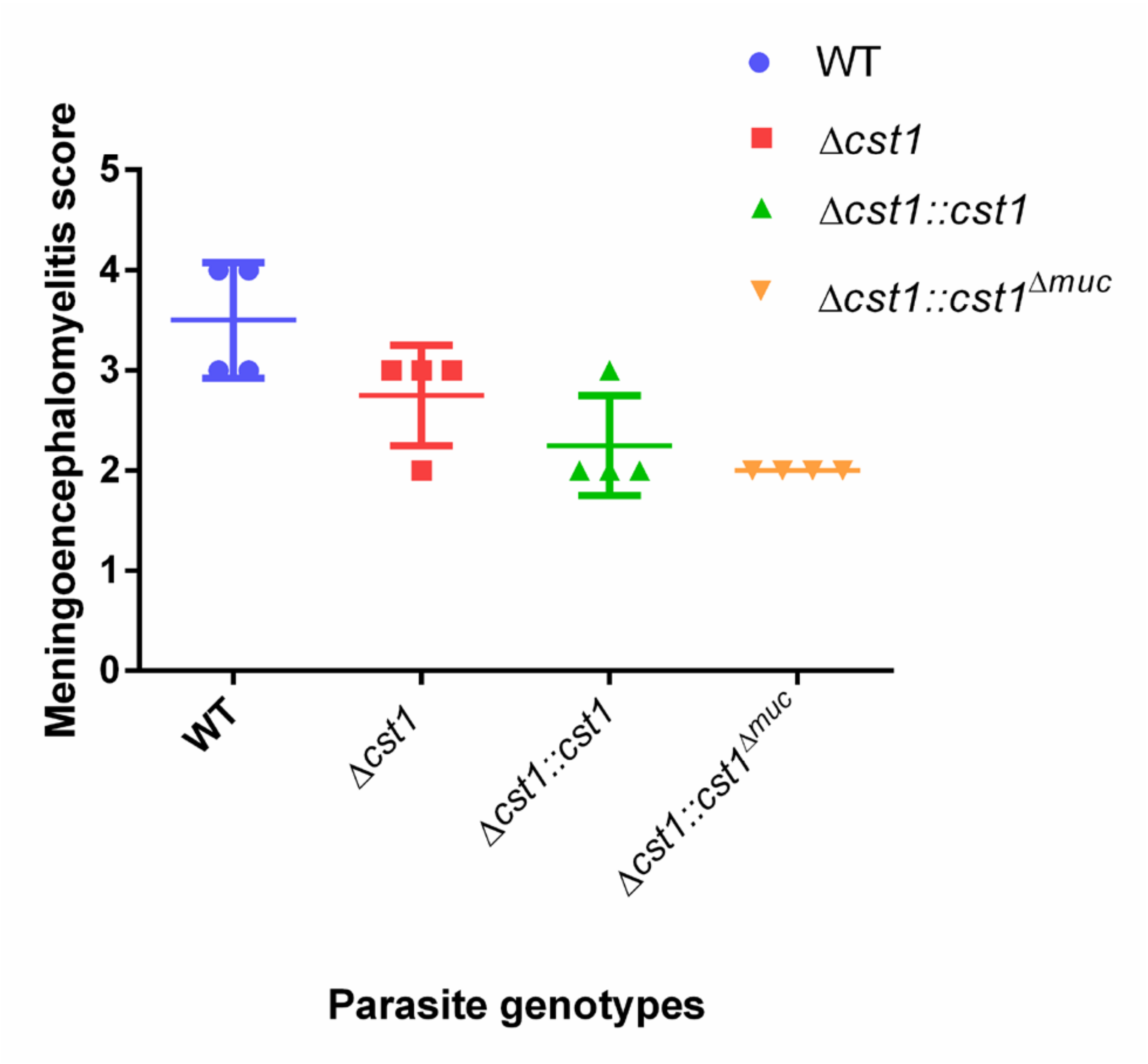

Figure S4. (B) Brains from mice infected with WT, Δ*cst1*, Δ*cst1*::*cst1*, or Δ*cst1*::*cst1*$^{\Delta muc}$ parasites for 4 weeks were sectioned and stained with H&E. Photomicrographs were obtained at 4X, 10X and 20X magnifications. Tissue cysts are indicated by a red arrow on 20X magnification images.

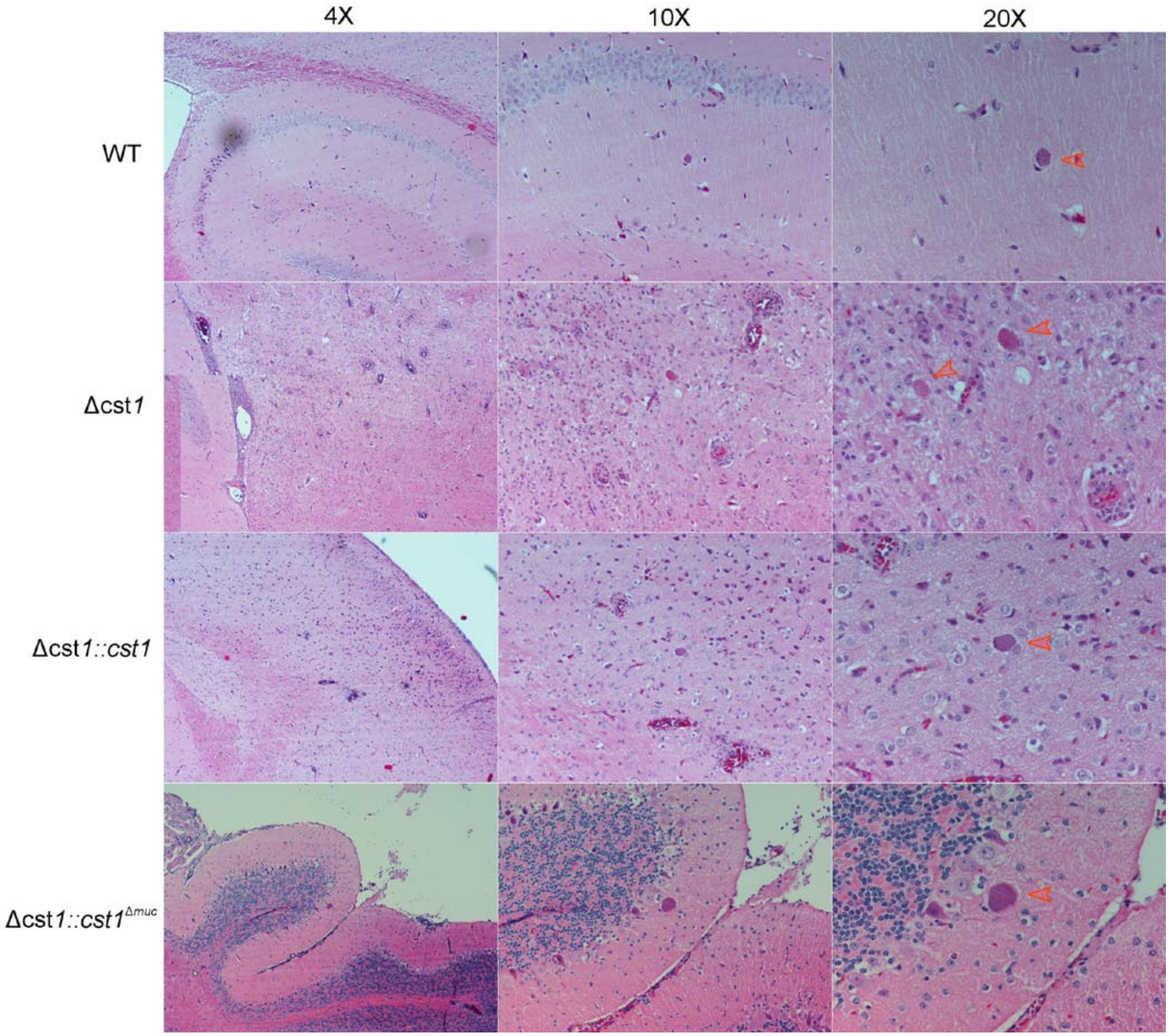

Supplement: Figure S4 — Histology of infected murine brains. (A) Hematoxylin and eosin (H&E) stained brain sections were scored for meningoencephalomyelitis using a scale of 0 to 5 (n = 4). p<0.05 WT vs Δcst1::cst1 Δmuc. (B) Brains from mice infected with WT, Δcst1, Δcst1::cst1, or Δcst1::cst1Δmuc parasites for 4 weeks were sectioned and stained with H&E. Photomicrographs were obtained at 4×, 10× and 20× magnifications. Tissue cysts are indicated by a red arrow on 20× magnification images. (PDF) [file ppat.1003823.s004.pdf]
