# Supplementary material for: The Toxoplasma gondii Cyst Wall Protein CST1 Is Critical for Cyst Wall Integrity and Promotes Bradyzoite Persistence
Source: PLoS Pathog. 2013 Dec 26;9(12):e1003823. doi: 10.1371/journal.ppat.1003823 (PMC3873430; doi:10.1371/journal.ppat.1003823)

Figure S5. Parasite growth measured at pH 7.0

A. Growth measurement using ^{3}H Uracil. Growth of WT (blue) or Δ*cst1* parasites (red) in HFF cells (10,000 parasites per well at time zero) at pH 7 was measured as ^{3}H-uracil incorporation into parasite DNA. Mean and standard deviation are shown. n = 3. This experiment was repeated 3 times and had similar results for wells harvested in triplicate.

B. Growth measurement by counting parasites. Growth of WT (blue), Δ*cst1* (red), Δ*cst1*::*cst1* (green), Δ*cst1*::*cst1*$^{\Delta muc}$ (yellow) parasites at pH7 culture condition was measured by lysing the host HFF cells with 0.5% saponin and counting the free parasites in a hemocytometer.

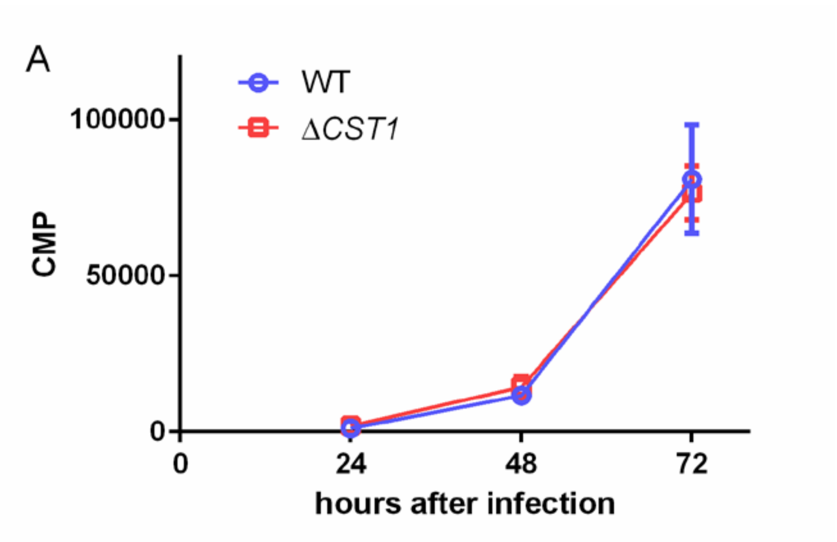

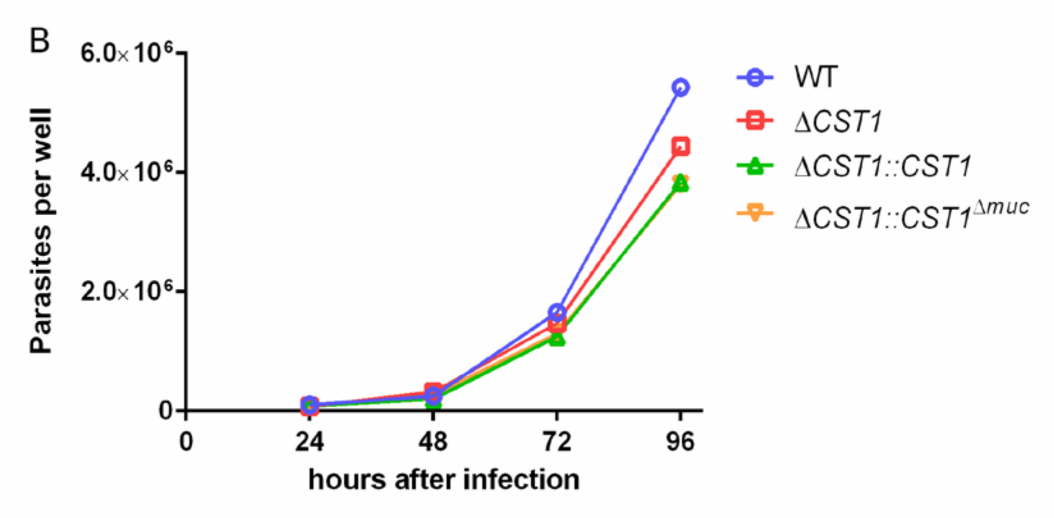

Supplement: Figure S5 — Parasite growth measured at pH 7.0. A. Growth measurement using 3H Uracil. Growth of WT (blue) or Δcst1 parasites (red) in HFF cells (10,000 parasites per well at time zero) at pH 7 was measured as 3H-uracil incorporation into parasite DNA. Mean and standard deviation are shown. n = 3. This experiment was repeated 3 times and had similar results for wells harvested in triplicate. B. Growth measurement by counting parasites. Growth of WT (blue), Δcst1 (red), Δcst1::cst1 (green), Δcst1::cst1Δmuc (yellow) parasites at pH 7 culture condition was measured by lysing the host HFF cells with 0.5% saponin and counting the free parasites in a hemocytometer. (PDF) [file ppat.1003823.s005.pdf]
