# Supplementary material for: The Toxoplasma gondii Cyst Wall Protein CST1 Is Critical for Cyst Wall Integrity and Promotes Bradyzoite Persistence
Source: PLoS Pathog. 2013 Dec 26;9(12):e1003823. doi: 10.1371/journal.ppat.1003823 (PMC3873430; doi:10.1371/journal.ppat.1003823)

Figure S6 Immunoblot using SalmonE and 73.18 to R5, ME49 and Pru strains of *T. gondii* and to ∆*cst1* and ∆*srs13* strains in KU80Pru *T. gondii*.

This demonstrates that mAb SalmonE binds to several type II strain *T. gondii* parasites and that a similar sized band is seen in these parasites. This immunoblot also demonstrates that monoclonal 73.18 binds to this high molecular weight band (recognized by mAb SalmonE) as well as a smaller band that we have identified as SRS13 (Weiss unpublished). The ∆SRS13 strain still reacts with mABSalmonE at 250 kDa but lacks the lower molecular weight bands.

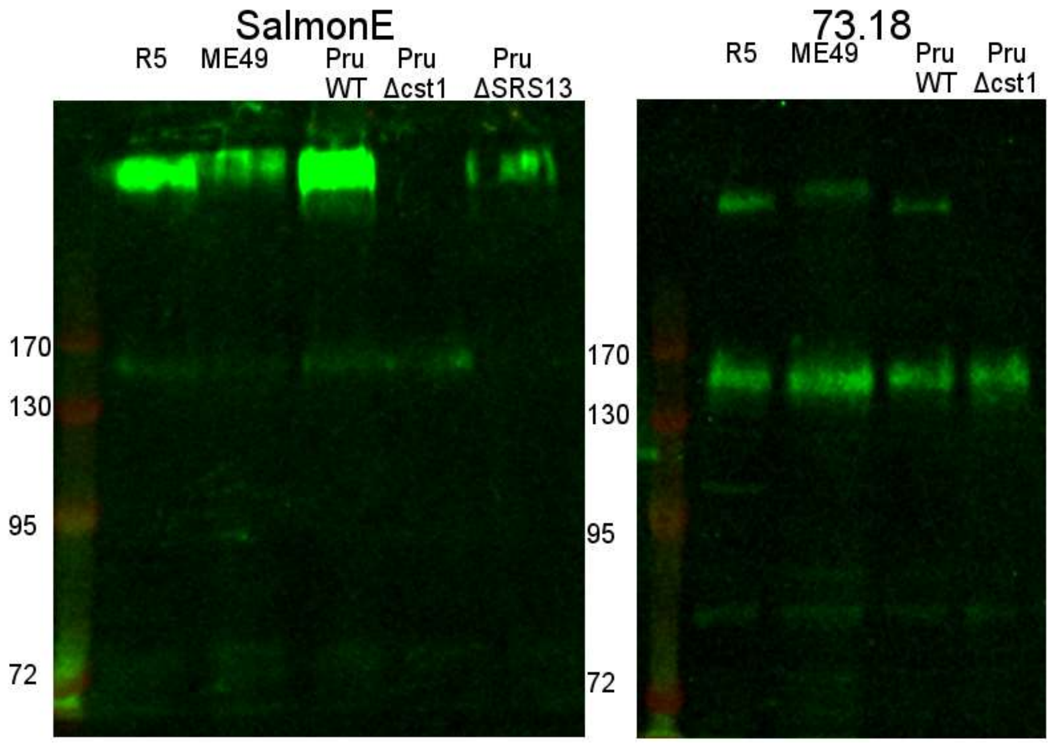

Supplement: Figure S6 — Immunoblot using SalmonE and 73.18 to R5, ME49 and Pru strains of T. gondii and to Δ cst1 and Δ srs13 strains in Pru Δku80 T. gondii . This demonstrates that mAb SalmonE binds to several type II strain T. gondii parasites and that a similar sized band is seen in these parasites. It also demonstrates that monoclonal 73.18 binds to this high molecular weight band (recognized by SalmonE) as well as a smaller band that we have identified as SRS13 (Tomita and Weiss unpublished). (PDF) [file ppat.1003823.s006.pdf]
