# Supplementary material for: The Toxoplasma gondii Cyst Wall Protein CST1 Is Critical for Cyst Wall Integrity and Promotes Bradyzoite Persistence
Source: PLoS Pathog. 2013 Dec 26;9(12):e1003823. doi: 10.1371/journal.ppat.1003823 (PMC3873430; doi:10.1371/journal.ppat.1003823)
Supplement: Table S1 — List of top 50 upregulated genes in bradyzoites. Gene up-regulation was determined by the RNA-seq RPKM value at pH 8 divided by that at pH 7. The genes are listed in order of highest upregulation. Gene ID and corresponding descriptions are from ToxoDB. (PDF) [file ppat.1003823.s007.pdf]

Table S1 List of top 50 upregulated genes in bradyzoites.

Gene up-regulation was determined by the RNA-seq RPKM value at pH 8 divided by that at pH 7. The genes are listed in order of highest upregulation. Gene ID and corresponding descriptions are from ToxoDB.

	Gene ID	Descriptions
1	TGME49_059020	bradyzoite antigen, putative
2	TGME49_007210	hypothetical protein
3	TGME49_068860	enolase 1
4	TGME49_009750	hypothetical protein
5	TGME49_009760	hypothetical protein
6	TGME49_007130	SRS49A (= SAG2Y)
7	TGME49_007160	SRS49D (= SAG2C)
8	TGME49_078080	hypothetical protein
9	TGME49_080570	bradyzoite surface antigen
10	TGME49_112320	hypothetical protein
11	TGME49_101170	SRS19D
12	TGME49_111370	methylmalonate-semialdehyde dehydrogenase, putative
13	TGME49_016140	ankyrin repeat-containing protein
14	TGME49_021840	hypothetical protein
15	TGME49_072220	tRNA-Gln
16	TGME49_052640	plasma-membrane H+-ATPase, putative
17	TGME49_094070	tRNA-Gly
18	TGME49_071320	hypothetical protein
19	TGME49_106230	hypothetical protein
20	TGME49_114250	hypothetical protein
21	TGME49_002110	hypothetical protein
22	TGME49_097990	tRNA-Pro
23	TGME49_031090	hypothetical protein
24	TGME49_093790	hypothetical protein
25	TGME49_002020	hypothetical protein
26	TGME49_055480	thioredoxin domain-containing protein
27	TGME49_120190	SRS16B (= SRS9)
28	TGME49_025290	nucleoside-triphosphatase, putative
29	TGME49_058510	cAMP-specific phosphodiesterase, putative
30	TGME49_007140	SRS49B (= SAG2X)
31	TGME49_059930	tRNA-Pro
32	TGME49_104920	hypothetical protein, conserved
33	TGME49_048920	tRNA-Met
34	TGME49_118680	3',5'--cyclic-nucleotide phosphodiesterase, putative
35	TGME49_024180	ankyrin repeat-containing protein
36	TGME49_073740	acetyl-CoA acyltransferase B, putative
37	TGME49_040930	molybdenum cofactor biosynthesis protein c, putative
38	TGME49_035860	subtilisin-like protease, putative
39	TGME49_062580	tRNA-Ile
40	TGME49_073940	hypothetical protein
41	TGME49_043470	hypothetical protein
42	TGME49_033880	tRNA-Ile
43	TGME49_062470	beta antigen, putative
44	TGME49_032460	tRNA-Asn
45	TGME49_104930	hypothetical protein
46	TGME49_013480	hypothetical protein
47	TGME49_078980	hypothetical protein, conserved
48	TGME49_090970	serine palmitoyltransferase, putative
49	TGME49_104940	hypothetical protein, conserved
50	TGME49_053330	Rhoptry kinase family protein, truncated (incomplete catalytic triad)
