# Supplementary material for: The Toxoplasma gondii Cyst Wall Protein CST1 Is Critical for Cyst Wall Integrity and Promotes Bradyzoite Persistence
Source: PLoS Pathog. 2013 Dec 26;9(12):e1003823. doi: 10.1371/journal.ppat.1003823 (PMC3873430; doi:10.1371/journal.ppat.1003823)
Supplement: Table S2 — Primers used for construction of plasmids for the deletion and complementation of CST1 . (PDF) [file ppat.1003823.s008.pdf]

Table S2. Primers used for construction of plasmids for the deletion and complementation of *CST1*.

Δ*cst1* plasmid (pRS416)

TGME49_064660 5' recombination sequence F/R
TTGGGTAACGCCAGGGTTTTCCCAGTCACGACGGTTTAAACATTATGTTGCTTACCCTATA
GCGGGTTTGAATGCAAGGTTTCGTGCTGATCAAACTAGTATCGACACAAGATGCCACGT

HXGPRT cassette F/R
ACTAGTTTGATCAGCACGA
TAACTAGTCCTCCACCGC

TGME49_064660 3' recombination sequence F/R
TTCTGGCAGGCTACAGTGACACCGCGGTGGAGGACTAGTTAGAGGCGCCGCCGCTACAG
GTGAGCGGATAACAATTTCACACAGGAAACAGCGCGGCCGCACATAAATCTGCAGAGCCTA

cDNA *CST1* complement plasmid (pSMART-BAC)

UPRT 5' recombination sequence F/R (1.1kb)
CAACTTGTCCACGTGGAATTGTTAACGTGAGCTCATGCTGGAGCTTCG
ATGGTACTCTACTTCGTTTAAGCTTTCCGCTCGCTGGGAC

CST1 5' upstream F/R (1.3kb)
TAAACGAAGTAGAGTACCATCATTACAATTCTGGG
TCAGATCTCGGGGTTCTTCCAGATCGAACCCGGA

CST1 cDNA F/R (7kb)
AACCCCGAGATCTGAGGCATGAGATGAAGAAAATAGAGGT
GCGGCGCCTCTACTCATGTCCGCAACAACAACAG

CST1 3' UTR (1.1kb)
GAGTAGAGGCGCCGCCGCTACAGTTTGTGGAATTTGAT
AGGGCGAAGGTTTTTGAAGACTTAGCTGCACCG

UPRT 3' recombination sequence F/R (1.1kb)
TCTTCAAAAACCTTCGCCCTGCTGTCTTGTCAGGTACT
CAAGTCACGTGAATTGTTAACTGGCGTTCGATCGACCGAAG

Mucin-null cDNA CST1 complement plasmid (pSMART-BAC)

Muc-null CST1 5' half R (6kb with CST1 cDNA F):
CTTATCGTCGTCATCCTTGTAATCTTCATGTTGCAGTGGGTC

Muc-null CST1 3' half F (300b with CST1 cDNA R):
GATTACAAGGATGACGACGATAAGCTCTTCGACTGGAGCAGT

Δ*cst1* construct genomic integration PCR primers F/R (1.3kb if integrated)

ACTGTATAGACGCTGGTTGTA
CCCGAGAAAAAGAAACGAACAGA

Primers for the detection of mutant CST1 (gDNA: 1824 bp, cDNA: 1546 bp, cDNAΔ*muc*: 781 bp)

CTTGATAAAACCTGCAAAAGCTGTG
GTCGATCTTAAAAGGCACCAAAAGA
